# Detection and quantification of natural *Wolbachia* in *Aedes aegypti* in Metropolitan Manila, Philippines using locally designed primers

**DOI:** 10.3389/fcimb.2024.1360438

**Published:** 2024-03-18

**Authors:** Jerica Isabel L. Reyes, Takahiro Suzuki, Yasutsugu Suzuki, Kozo Watanabe

**Affiliations:** ^1^ Molecular Ecology and Health Laboratory, Center for Marine Environmental Studies (CMES), Ehime University, Matsuyama, Japan; ^2^ Graduate School of Science and Engineering, Ehime University, Matsuyama, Japan

**Keywords:** Wolbachia, aedes aegypti, vector control, arbovirus, mosquito

## Abstract

**Background:**

The Philippines bears health and economic burden caused by high dengue cases annually. Presently, the Philippines still lack an effective and sustainable vector management. The use of *Wolbachia*, a maternally transmitted bacterium, that mitigate arbovirus transmission has been recommended. Cytoplasmic incompatibility and viral blocking, two characteristics that make *Wolbachia* suitable for vector control, depend on infection prevalence and density. There are no current *Wolbachia* release programs in the Philippines, and studies regarding the safety of this intervention. Here, we screened for *Wolbachia* in *Aedes aegypti* collected from Metropolitan Manila, Philippines. We designed location-specific primers for qPCR to test whether this improved *Wolbachia* detection in *Ae. aegypti.* We explored if host sex and *Wolbachia* strain could be potential factors affecting *Wolbachia* density.

**Methods:**

*Ae. aegypti* mosquitoes (n=429) were screened for natural *Wolbachia* by taqman qPCR using location-specific *Wolbachia* surface protein primers (*wsp*AAML) and known *16S rRNA* primers. Samples positive for *wsp*AAML (n=267) were processed for Sanger sequencing. We constructed a phylogenetic tree using IQ-TREE 2 to further characterize *Wolbachia* present in the Philippine *Ae. aegypti*. We then compared *Wolbachia* densities between *Wolbachia* groups and host sex. Statistical analyses were done using GraphPad Prism 9.0.

**Results:**

*Wolbachia* prevalence for 16S rRNA (40%) and *wsp*AAML (62%) markers were high. *Wolbachia* relative densities for 16S rRNA ranged from −3.84 to 2.71 and *wsp*AAML from −4.02 to 1.81. Densities were higher in male than female mosquitoes. *Wolbachia* strains detected in *Ae. aegypti* clustered into supergroup B. Some 54% (123/226) of these sequences clustered under a group referred to here as “wAegML,” that belongs to the supergroup B, which had a significantly lower density than wAegB/*w*AlbB, and *w*AlbA strains.

**Conclusion:**

Location-specific primers improved detection of natural *Wolbachia* in *Ae. aegypti* and allowed for relative quantification. *Wolbachia* density is relatively low, and differed between host sexes and *Wolbachia* strains. An economical way of confirming sporadic or transient *Wolbachia* in *Ae. aegypti* is necessary while considering host sex and bacterial strain.

## 1 Introduction

The Philippines continues to experience the health burden caused by high dengue cases, ranking number one in Asia with 17,630 deaths last March 2021 (Edillo et al., 2015, 2022; Ong et al., 2022). The persistent high cases annually prompted the Philippine government to establish the National Dengue Prevention and Control Program with vector surveillance and management as one of the key targets (Dengue Prevention and Control Program | Department of Health website, n.d.). *Aedes aegypti* mosquito is the primary vector of Dengue in the Philippines owing to its adaptability in changing environments (Edillo et al., 2022). To mitigate infections, recommendations for vector management including the reduction of breeding sites, and improvement of water systems have been proposed. More importantly, Ong et al. (2022) highlighted the need for a more sustainable approach involving the use of the bacterium *Wolbachia* (Ong et al., 2022). Currently, the Philippines still lacks local studies that could provide a baseline information for assisting future mass release implementations (Ong et al., 2022).

*Wolbachia* was discovered in *Culex pipiens* (Hertig and Wolbach, 1924) and may induce cytoplasmic incompatibility (CI), wherein gametes fail to produce viable offspring owing to incompatible *Wolbachia* infection (Yen and Barr, 1973). *Wolbachia* also naturally infect arthropods such as *Drosophila* spp (Min and Benzer, 1997; Brownlie et al., 2009; McMeniman et al., 2009; Osborne et al., 2009), *Anopheles* spp (Walker et al., 2021), and *Aedes albopictus* (Dutton and Sinkins, 2004; Yang et al., 2021). Different *Wolbachia* strains, *i.e. w*Melpop, *w*Mel, and *w*AlbB, from these natural hosts can exert life-shortening effects (Min and Benzer, 1997; McMeniman et al., 2009), or confer nutritional benefits (Brownlie et al., 2009), efficient maternal transmission (Liu and Li, 2021), and antiviral protection (Osborne et al., 2009; Liu and Li, 2021; Reyes et al., 2021). CI, maternal transmission, and the antiviral effects of *Wolbachia* against arthropod-borne viruses all contribute to the efficiency of the mass release of *Wolbachia*-transinfected *Ae. aegypti* (Indriani et al., 2020; Ahmad et al., 2021). Currently, only *D. melanogaster*-derived *w*Mel and *Ae. albopictus*-derived *w*AlbB are used in release programs (Indriani et al., 2020; Ahmad et al., 2021; Ross et al., 2022). Release programs can either result in mosquito population suppression or replacement (Ross et al., 2020). The former involves the release of *Wolbachia*-infected males that induce CI with wild female mosquitoes, whereas both CI-inducing male mosquitoes and females carrying a pathogen-blocking strain that is maternally transmitted are released in the latter (Ross et al., 2020).

Unlike other host species, studies on *Ae. aegypti* have mostly revealed the absence of natural *Wolbachia* (Gloria-Soria et al., 2018; Goindin et al., 2018). Novel *Wolbachia* transinfection in mosquitoes facilitates population suppression and replacement (Ross et al., 2020). However, the occasional presence of *Wolbachia* in *Ae. aegypti* populations without any preceding *Wolbachia* have been reported. These were found in the USA (Coon et al., 2016), Mexico (Kulkarni et al., 2019), Panama (Bennett et al., 2019), India (Balaji et al., 2019), Malaysia (Teo et al., 2017), Thailand (Thongsripong et al., 2018), China (Zhang et al., 2022), Taiwan (Chao and Shih, 2023), and Philippines (Carvajal et al., 2019; Regilme et al., 2021; Muharromah et al., 2023). Thus, understanding biological factors that may influence the variable presence of natural *Wolbachia* in *Ae. aegypti* is necessary. More so, finding an economical way to improve detection of *Wolbachia* could help initial surveillance in countries where mass release programs could be implemented. This is particularly important in low-income countries like the Philippines, gravely affected by arboviral diseases.

Natural *Wolbachia* hosts are characterized by consistently high *Wolbachia* prevalence (Inácio da Silva et al., 2021). *Culex pipiens*, *Culex quinquefasciatus*, *Ae. albopictus*, *Anopheles moucheti*, and *Anopheles demeilloni* can exhibit 90% to 100% *Wolbachia* prevalence (Bergman and Hesson, 2021; Inácio da Silva et al., 2021). In *Anopheles gambiae* and *Ae. aegypti*, prevalence is relatively low and can vary from 8–24% and 4.3–58%, respectively (Inácio da Silva et al., 2021). The detection of natural *Wolbachia* in *Ae. aegypti* remains inconclusive. The reported presence of *Wolbachia* in the said mosquito host seems to be sporadic, transient, and low in prevalence making it difficult to quantify. Thus, more studies regarding prevalence, biological factors, and mechanisms are needed to understand the nature of natural *Wolbachia* sporadically found in different populations of *Ae. aegypti.*

In general, *Wolbachia* prevalence and density influence maternal transmission fidelity and pathogen blocking extent (Ikeda et al., 2003; Unckless et al., 2009; Hoffmann et al., 2014; López-Madrigal and Duarte, 2019; Liu and Li, 2021; Shropshire et al., 2021; Walker et al., 2021). Achieving high and stable *Wolbachia* introgression into communities via transinfected *Ae. aegypti* depends on the number of mosquitoes that become infected with the endosymbiont (Indriani et al., 2020; Ahmad et al., 2021). Other hosts like *Drosophila* spp. and the moth *Ephestia kuehniella* exhibit varying CI levels relative to *Wolbachia* density; i.e., mostly high CI with high density (Hoffmann et al., 1986; Bourtzis et al., 1996; Ikeda et al., 2003; Unckless et al., 2009; Turelli et al., 2018). However, one study found that high *w*Mel density in *Drosophila simulans* did not translate into elevated CI but strengthened host immune expression (Shropshire et al., 2021). Density also has been linked to virus inhibition (Bian et al., 2010; Frentiu et al., 2010; Lu et al., 2020). For example, higher *w*AlbB and *w*Melpop-CLA densities in *Ae. albopictus* was correlated with stronger blocking *in vitro* (Frentiu et al., 2010; Lu et al., 2020). Likewise, *Ae. aegypti* mosquitoes transinfected with *w*AlbB inhibited dengue replication and transmission by regulating immunity and longevity, respectively (Bian et al., 2010).

*Wolbachia* density is host sex-dependent. Density is reported higher in adult female than male hosts, as observed in *D. simulans* as well as the planthoppers *Laodelphax striatellus* and *Sogatella furcifera* (Noda et al., 2001; Correa and Ballard, 2012)*. Wolbachia* densities are also more stable in female than male insect hosts and decline with age in males (Noda et al., 2001; Unckless et al., 2009; Correa and Ballard, 2012). However, males exhibit higher *Wolbachia* density than females in some insects such as *Diaphorina citri* (Hoffmann et al., 2014). *Wolbachia* density and prevalence in host sex also vary with strain in *Ae. albopictus* (Tortosa et al., 2010; Yang et al., 2022). *w*AlbB density was higher in males than females, whereas *w*AlbA density was higher in females than males. *Wolbachia w*AlbA density in male *Ae. albopictus* also decreased with age (Tortosa et al., 2010). Further, a higher prevalence of *w*AlbA and *w*AlbB coinfection was observed in females than males, in which infection prevalence varied according to *w*Alb strain (Tortosa et al., 2010; Yang et al., 2022).

*Wolbachia* density is also determined by *Wolbachia* strain. Different *Wolbachia* strains vary in density (Dutton and Sinkins, 2004; Hu et al., 2020), viral inhibition (Osborne et al., 2009; Martinez et al., 2017), and CI (Liang et al., 2020) strength in hosts. For instance, *w*AlbB resides in *Ae. albopictus* at a higher density than *w*AlbA (Dutton and Sinkins, 2004; Hu et al., 2020). Further, *Wolbachia* strains *w*Mel, *w*Au, *w*Ri, and *w*No that exist at high density in *D. simulans* confer viral protection, unlike *w*Ha (Osborne et al., 2009). Lastly, strains *w*Melpop, *w*Mel, and *w*AlbB exhibit stable and high-density infection in transinfected *Ae. aegypti*, despite being transferred from their natural hosts *D. melanogaster* and *Ae. albopictus* (McMeniman and O’Neill, 2010; Fraser et al., 2017; Ross, 2021).

Currently, information on the presence of natural *Wolbachia* in *Ae. aegypti* varies and knowledge on density in relation to biological factors is limited (Teo et al., 2017; Bennett et al., 2019; Carvajal et al., 2019). Detection of *Wolbachia* also differs among states and cities within a country (Chen et al., 2016; Schuler et al., 2018; Zhang et al., 2022). Previous studies on prevalence have used general primers based on *Wolbachia* sequences from established natural hosts (Teo et al., 2017; Bennett et al., 2019; Carvajal et al., 2019; Kulkarni et al., 2019); thus primers currently used may not detect rare and low-density strains (Marcon et al., 2011; Simões et al., 2011). These limitations suggest that validation methods are necessary to perform initial surveillance targeting specific local *Wolbachia* populations. Lastly, *Wolbachia* density in naturally infected hosts is regulated by multiple factors; e.g., host genotype (Mouton et al., 2006), host sex (Tortosa et al., 2010; Mejia et al., 2022), and *Wolbachia* strain (Dutton and Sinkins, 2004; Hu et al., 2020). However, no study has clarified the biological factors that affect *Wolbachia* density in *Ae. aegypti.*

The present study aims to investigate if locally designed primers for qPCR could help validate the presence and density of natural *Wolbachia* in local mosquito populations. We also determined if density could be influenced by host sex and *Wolbachia* strain. We did this by utilizing locally designed *Wolbachia* surface protein (*wsp*) primers suitable for *Ae. aegypti* collected from Metro Manila, Philippines. We then used these primers to quantify *Wolbachia* density in 429 individual mosquitoes and compared against a known general primer used for conventional PCR. Next, we examined *Wolbachia* density between *Wolbachia* host sex and strains in *Ae. aegypti* populations. We hypothesized that the presence of sporadic, low density natural *Wolbachia* in *Ae. aegypti* can be detected and quantified by using primers designed for local mosquito populations and that *Wolbachia* density differs depending on *Wolbachia* host sex and strain. An economical way to conduct initial surveillance of *Wolbachia* in *Ae. aegypti* is necessary especially in low-income countries like the Philippines, where an effective vector control strategy is needed.

## 2 Materials and methods

### 2.1 Mosquito sample collection

We used DNA samples extracted from *Ae. aegypti* adult mosquitoes collected in the National Capital Region (Manila) of the Philippines; these samples were previously used for *Wolbachia* detection via conventional PCR (Carvajal et al., 2019) and ddRAD-Seq (Muharromah et al., 2023). *Wolbachia* was detected in 11.9% (80/672) of these samples by PCR (Carvajal et al., 2019) and sequence reads of 26,299 (*w*AlbA) and 43,778 (*w*AlbB) were mapped across the entire *Wolbachia* genome out of 146,239,637 filtered reads obtained by ddRAD-Seq (Muharromah et al., 2023). Thus, the samples were considered suitable for validating primer incompatibility. Each individual mosquito was previously screened and processed for DNA extraction as described by Carvajal et al. (2019). The total DNA of individual mosquitoes was extracted using a Blood and Tissue DNEasy Kit (Qiagen, Hilden, Germany) according to the manufacturer’s protocol with slight modifications (Crane, 2013). All samples were stored as DNA at −80°C for long term preservation. Of 672 individual mosquitoes used by Carvajal et al. (2019), we selected 429 samples based on sufficient volume and DNA concentration for downstream assays.

### 2.2 *Wolbachia* detection via conventional PCR

Data on conventional PCR results were obtained from the study of Carvajal et al. (2019) (**Supplementary Data**) and used as a baseline reference in the present study in relation to *Wolbachia* prevalence and density detected with the newly designed primers. Briefly, Carvajal et al. (2019) used two known markers targeting the *16S rRNA* gene, which has a slow evolutionary rate, and another marker targeting the highly variable *Wolbachia* surface protein (*wsp*), which is suitable for strain identification (O’Neill et al., 1992; Zhou et al., 1998). The sequences of *16S rRNA* and *wsp Wolbachia*-specific primers were as follows: *Wspecf* (5′-GAA GAT AAT GAC GGT ACT CAC-3′) and *Wspecr* (5′-AGC TTC GAG TGA AAC CAA TTC-3′) (O’Neill et al., 1992); *wsp* 81F (5′-TGG TCC AAT AAG TGA TGA AGA AAC-3′) and *wsp* 691R (5′-AAA AAT TAA ACG CTA CTC CA-3′) (Zhou et al., 1998). PCR thermocycling conditions were conducted according to the published protocol (Carvajal et al., 2019).

### 2.3 Primer design for *wsp* based on local *wsp* sequences

Most *wsp* primers designed for *Wolbachia* detection are strain-specific (Zhou et al., 1998; Dutton and Sinkins, 2004). Thus, we developed new primers specific for our local samples to consider for primer incompatibility and to quantify *Wolbachia* density. First, we obtained 118 *wsp* sequences from the *Ae. aegypti* samples of Carvajal et al. (2019) (GenBank popset 1712729902). Next, a multiple sequence alignment was performed using MUSCLE, and the results were visualized in Codon Code Aligner version 1.2.4 (https://www.codoncode.com/aligner/). The consensus sequence produced from the alignment was then inputted into Primer-BLAST (Ye et al., 2012) to design *wsp* primers targeting the *Ae. aegypti* samples. Primer-BLAST generated five primer pairs (**Supplementary Table 1**), which were first validated via gradient conventional PCR using a known *Cx. quinquefasciatus* positive sample. Among the primer pairs, primers *wsp*AAML 01 and *wsp*AAML 05 were selected for further optimization because they exhibited the correct band size of target markers without nonspecific binding in the sample (**Supplementary Figure 1**). To select the most suitable *wsp*AAML primer pair for downstream analysis, we determined the optimized annealing temperature and primer concentration for both pairs (**Supplementary Figure 2**). We selected *wsp*AAML 05, given that its PCR efficiency (**Supplementary Figure 3**) fell within the standard MIQE guideline of ≥90% (Bustin et al., 2009).

### 2.4 Natural *Wolbachia* infection validation using TaqMan qPCR and strain identification via sequencing

To quantify *Wolbachia* density, Taqman qPCR targeting both *16S rRNA* and *wsp*AAML was conducted. *Wolbachia* quantification was performed using a well-established primer set targeting the *16S rRNA* marker (*16S*F 5′-AGT GAA GA A GGC CTT TGG G-3′; *16S*R 5′-CAC GGA GTT AGC CAG GAC TTC-3′) but with a modification of the fluorescent dye of the probe (Fraser et al., 2020). Instead of LC640, we used TET as the reporter dye and BHQ1 as its quencher (5′TET-CTG TGA GTA CCG TCA TTA TCT TCC TCA CT-BHQ13′). *Wolbachia* was also confirmed using the newly designed *Wolbachia* surface protein (*wsp*) primers (*wsp*AAML F 5′-AGC ATC TTT TAT GGC TGGT GG-3′; wspAAML R 5′- AAT GCT GCC ACA CTG TTT GC-3′; *wsp*AAML probe 5′FAM-ACG ACG TTG GTG GTG CAA CAT TTG C-TAMRA3′) with the *Ae. aegypti* ribosomal protein S17 (*RPS17*) gene as a reference gene (*17S*F 5′-TCC GTG GTA TCT CCA TCA AGC T-3′; *17S*R 5′- CAC TTC CGG CAC GTA GTT GTC-3′; 17S probe 5′HEX- CAG GAG GAG GAA CGT GAG CGC AG-BHQ13′) (Frentiu et al., 2010). In total, 429 individual mosquitoes were screened for the presence of *Wolbachia* using qPCR with a cut-off Cq value of 35. The cut-off value was set from a qPCR experiment of *Cx. quinquefasciatus* samples representing true natural *Wolbachia* infection and three replicates of the no-template control for each target gene; negative detections of no template controls were confirmed using gel electrophoresis showing no bands, hence no true amplification (**Supplemental Figure 4**).

All singleplex PCR reactions were performed in a final volume of 10 µl containing 5 µl of 2x iTaq Universal Probes Supermix (Bio Rad) with 0.3 µM RPS17 primers, 0.2 µM 16S rRNA primers, or 0.5 µM wsp primers and 0.2 µM, 0.15 µM, or 0.3 µM of their corresponding TaqMan probes, respectively, with nuclease-free water added to reach the final volume. The following thermal profile was used for both *RPS17* and *16S rRNA* with a CFX96 touch deep well real-time PCR detection system (Bio-Rad Tokyo, Japan): an initial polymerase activation at 95°C for 30 s followed by 40 cycles of denaturation at 95°C for 5 s, and combined annealing/extension at 60°C for 10 s. The *wsp* thermal profile included an initial polymerase activation at 95°C for 2 min followed by 40 cycles of denaturation at 95°C for 5 s, and combined annealing/extension at 58.8°C for 30 s. Each PCR amplification included a *Wolbachia*-infected *Cx. quinquefasciatus* positive control and a no-template control. *Wolbachia* density was derived from the Cq values expressed as the relative density of wspAAML normalized to single-copy *RPS17* (Livak and Schmittgen, 2001). This was used to compare density between host sex and *Wolbachia* strains. Relative densities reported in this study were log-transformed (log10). Following detection, qPCR-confirmed *wsp*AAML-positive products were cleaned using a mixture of alkaline phosphatase (TaKaRa) and exonuclease I (TaKaRa). The cleaned samples were subjected to Sanger sequencing for strain identification.

### 2.5 *Wolbachia* phylogeny

*Wolbachia* phylogeny was inferred using the maximum-likelihood criterion. For this analysis, we used *Ae. aegypti* samples in which the *wsp* gene was detected via qPCR (n = 226). We also obtained additional *wsp* sequences from other host species, e.g., *Aedes* spp., *Anopheles* spp., *Culex* spp., and others indicated as reference sequences (n = 511), from NCBI GenBank (**Table 1**). In total, 737 wsp sequences were aligned using MUSCLE in CodonCode Aligner version 1.2.4 (https://www.codoncode.com/aligner/) and then trimmed to a final length of 103 nucleotide bases. The sequences containing 103 nucleotide bases correspond to the homologous region across all sequences. Using DNASp version 6.12.03 (Rozas et al., 2017), we obtained 102 haplotypes, and we subjected the representative sequences of each haplotype to phylogenetic analysis. Tree reconstruction was conducted using only *wsp* given the high evolutionary rate of the gene; i.e., its suitability for strain identification (Zhou et al., 1998; Ren et al., 2020). IQ-TREE 2 (http://iqtree.org) (Minh et al., 2020) was used where the appropriate substitution model was first identified through ModelFinder implemented as a function of the software (Kalyaanamoorthy et al., 2017), from which TPM2+G4 was selected as the best-fit model. We set the ultrafast bootstrap approximation (UFBoot) in IQ-TREE to 1,000 iterations, the minimum correlation coefficient to 0.99, and the other parameters to their default settings (Hoang et al., 2018). For visualization and annotation, we used iTOL (https://itol.embl.de/) (Letunic and Bork, 2021).

**Table 1:** Reference sequences for *Wolbachia* supergroups.

Supergroup	Group	*Wolbachia* host species	GenBank No.
A	*w*Mel	*Drosophila melanogaster*	AF020072
	wAlbA	*Aedes albopictus*	AF020058
	wMors	*Glossina morsitans*	AF020079
	wRi	*Drosophila simulans* (Riverside)	AF020070
	wUni	*Muscidifurax uniraptor*	AF020071
	wHa	*Drosophila sechellia*	AF020068
	wPap	*Phlebotomus papatasi*	AF020082
B	wPip	*Culex pipiens*	AF020061
	*w*Pip	*Culex quinquefasciatus*	AF020060
	wAlbB	*Aedes albopictus*	AF020059
	wAegB	*Aedes aegypti*	MF999264
	wMa	*Drosophila simulans*	AF020069
Outgroup		*Brugia pahangi*	AY527207

The table provides the different reference sequences used for phylogenetic analysis. Each sequence represents a specific host and supergroup.

### 2.6 Statistical analysis

Mann Whitney tests were used to determine statistical differences between *Wolbachia* densities of male and female mosquitoes as well as those between the densities of samples detected as positive or negative using conventional PCR. To compare densities between *Wolbachia* strains, Dunn’s multiple comparison test was performed. Statistical calculations were conducted in GraphPad Prism version 9.2.0 for Windows (www.graphpad.com), and p -values of ≤0.01 or ≤0.0001 were considered statistically significant.

## 3 Results

### 3.1 Validation of natural *Wolbachia* infection in *Ae. aegypti*

Screening of *Wolbachia* in *Ae. aegypti* using *16S rRNA* and *wsp* qPCR revealed an overall prevalence of 40% (172/429) and 62% (267/429) in the mosquito population, respectively (**Table 2**). *Wolbachia* density expressed in logarithmic scale refers to the relative abundance of the target gene normalized to the reference gene, *RPS17* (Livak and Schmittgen, 2001). Thus, the median relative *Wolbachia* density was −1.99 and −2.09 for *16S rRNA* and *wsp*AAML qPCR assays, respectively. The relative *Wolbachia* densities of *16S rRNA*- and *wsp*AAML-positive samples ranged from −3.84 to 2.17 and −4.02 to 1.81, respectively.

**Table 2:** Infection prevalence of natural *Wolbachia* in *Ae. aegypti* based on conventional PCR (cPCR) and qPCR.

	*Wolbachia*-positive/total (% positive)					
	*16S rRNA*			*wsp*		
	Female	Male	Total	Female	Male	Total
**cPCR**	23/429 (5.4)	21/429 (4.9)	44/429 (10.3)	28/429 (6.5)	29/429 (6.8)	57/429 (13.3)
**qPCR**	103/429 (24.0)	69/429 (16.0)	172/429 (40.0)	158/429 (36.8)	109/429 (25.4)	267/429 (62.2)

The table shows a comparison of Wolbachia-positive samples detected using conventional PCR and qPCR.

Comparing our qPCR results with the conventional PCR results of Carvajal et al. (2019), we found that 91% (40/44) of the mosquitoes positive for 16S rRNA in conventional PCR showed the same result in our qPCR, whereas only 9% equivalent to 4 out of 44 of the mosquito samples previously detected as positive by standard PCR were detected as negative via qPCR. Of the 385 samples confirmed as negative for 16S rRNA via conventional PCR, 34% (132/385) were positive according to qPCR, whereas 66% (253/385) were consistent with negative conventional PCR detection. Regarding *wsp*, 100% (57/57) of mosquito samples that were positive according to conventional PCR were also positive in our qPCR. Conventional PCR *wsp*-negative samples exhibited an infection prevalence of 57% positive (213/372) and 43% negative (159/372) detection according to qPCR.

**Figure 1:**
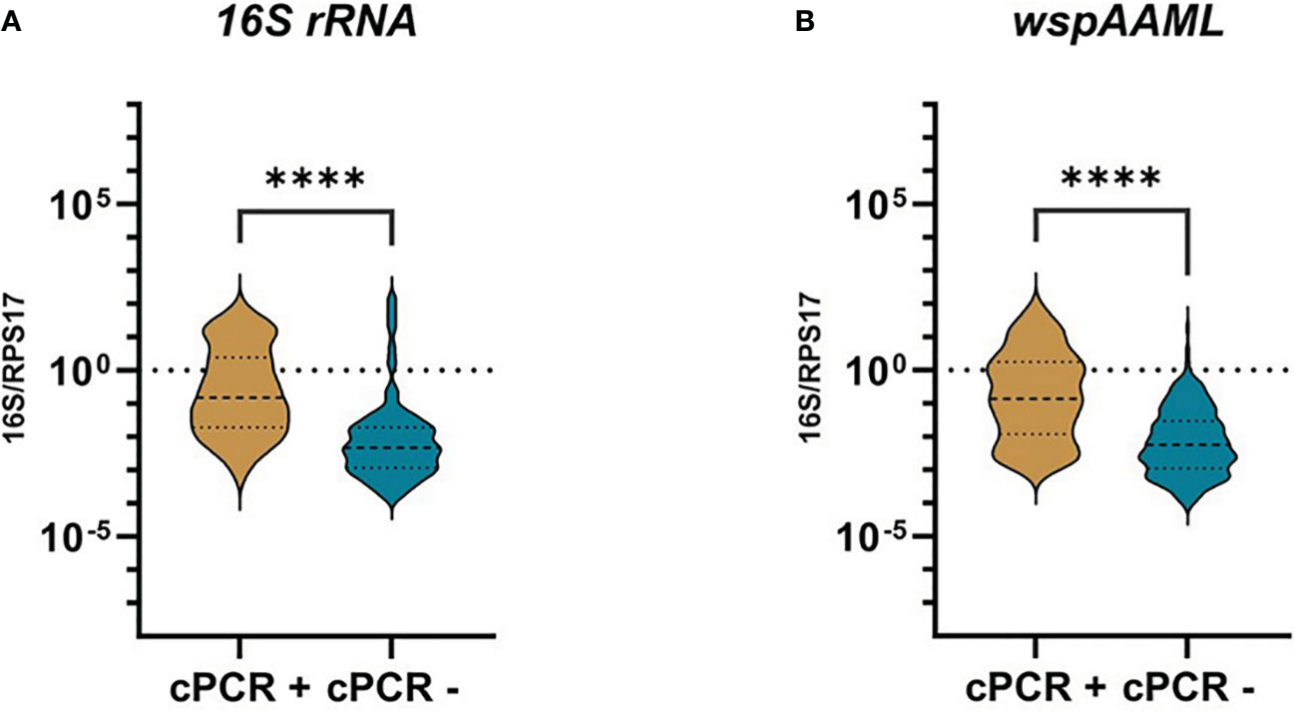
*Wolbachia* density of qPCR-positive *Ae. aegypti* grouped according to conventional PCR results. Individual mosquitoes detected as either positive for *16S rRNA* (n =172) or *wspAAML* (n = 267) via qPCR were grouped based on *Wolbachia* detection results according to conventional PCR (cPCR). Relative *Wolbachia* density is expressed as the ratio of the target gene to single-copy *RPS17* in logarithmic scale. **(A)** Using *16S rRNA* primers, cPCR positive (n = 40) and negative (n = 132) samples had a median relative *Wolbachia* density of −0.8250 and −2.325, respectively. **(B)** Using *wspAAML* primers, cPCR positive (n = 54) and negative (n = 213) samples had a median relative *Wolbachia* density of −0.8550 and −2.250, respectively. **** indicates a significant difference between cPCR-positive and cPCR-negative *Ae. aegypti* at *p ≤ 0.0001.*.

In an attempt to explain the contrasting negative conventional PCR and positive qPCR *Wolbachia* detection results, we compared the relative *Wolbachia* densities expressed in logarithmic scales, between samples found to be either *Wolbachia*-negative or positive via conventional PCR by Carvajal et al. (2019) (**Figure 1**). We found an approximately 30-fold higher relative *Wolbachia* density in *16S rRNA* (median = −0.8250) and *wsp*AAML (median = −0.8550) *Wolbachia*-positive mosquitoes compared with the conventional PCR-negative samples for *16S rRNA* (p < 0.001; median = −2.325) and *wsp* (p < 0.001; median = −2.250). Finally, it is worth mentioning that in qPCR alone, only 21% (91/429) of samples were positive for both *16S rRNA* and *wsp*AAML markers.

### 3.2 Natural *Wolbachia* density differs according to host sex

**Figure 2:**
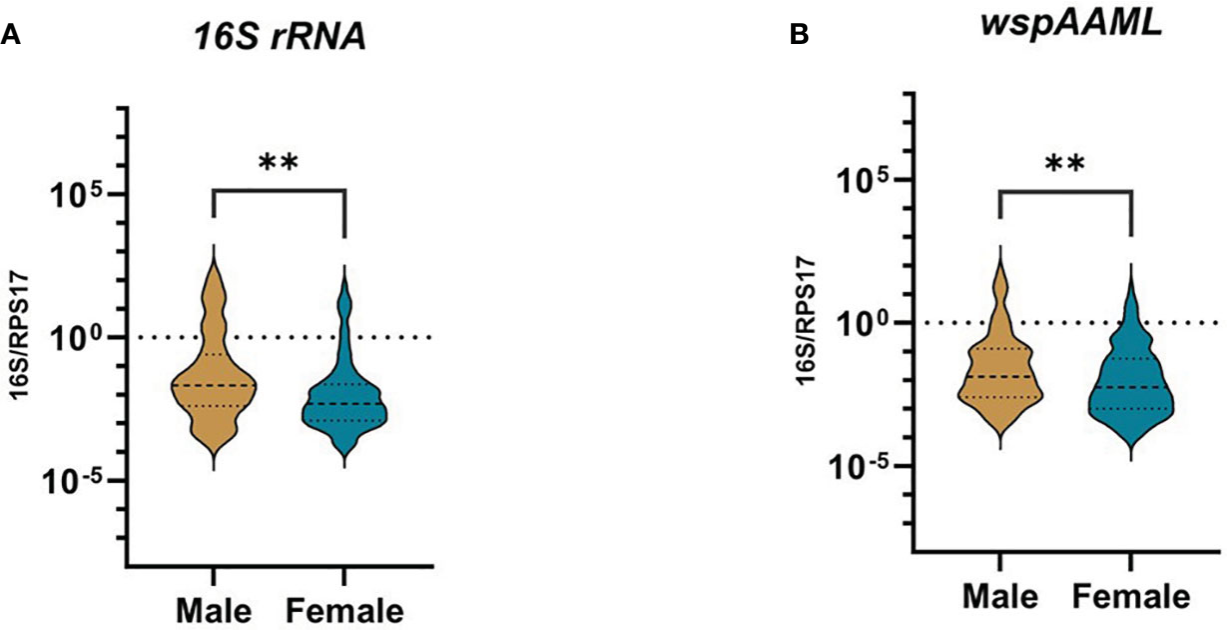
Presence of natural *Wolbachia* between male and female *Ae. aegypti.* Mosquitoes that were *Wolbachia-*positive according to TaqMan qPCR were classified as either male (*16S* r*RNA* = 69, *wspAAML =* 109) or female (*16S* r*RNA* = 103, *wspAAML =* 158). Relative *Wolbachia* density is shown as the ratio of the target gene to *RPS17* in logarithmic scale. The 95% confidence interval of the median is indicated by the blue and violet lines. Relative densities are shown for **(A)** *16S rRNA* and **(B)** *wspAAML* markers. In both **(A, B)**, *Wolbachia* density differed significantly according to host sex. **p ≤ 0.01.

For both *16S rRNA* and *wsp*AAML qPCR assays, *Wolbachia* densities were ≥10-fold higher in male than female *Ae. aegypti* (p ≤ 0.01 for both; **Figure 2**). In the *16S rRNA* marker, male mosquitoes (n = 69) exhibited relative *Wolbachia* densities between −3.57 and 2.17 with a median value of −1.67, whereas females (n = 103) exhibited a relative *Wolbachia* density range from −3.84 to 1.82 and a median value of −2.31. Regarding the *wsp*AAML marker, male mosquitoes (n = 109) exhibited relative *Wolbachia* densities between −3.53 and 1.81 with a median value of −1.88, whereas females (n = 158) exhibited a range from −4.02 to 1.28 and a median value of −2.24. Although *Wolbachia* density was higher in male *Ae. aegypti* than in females, the prevalence of infection was higher among females (*16S rRNA* = 24%, *wsp*AAML = 36.8%) than males (*16S rRNA* = 16%, *wsp*AAML = 25%), regardless of the marker used for detection via qPCR (**Table 2**).

### 3.3 Phylogenetic analysis of *Wolbachia* strains in *Ae. aegypti*

**Figure 3:**
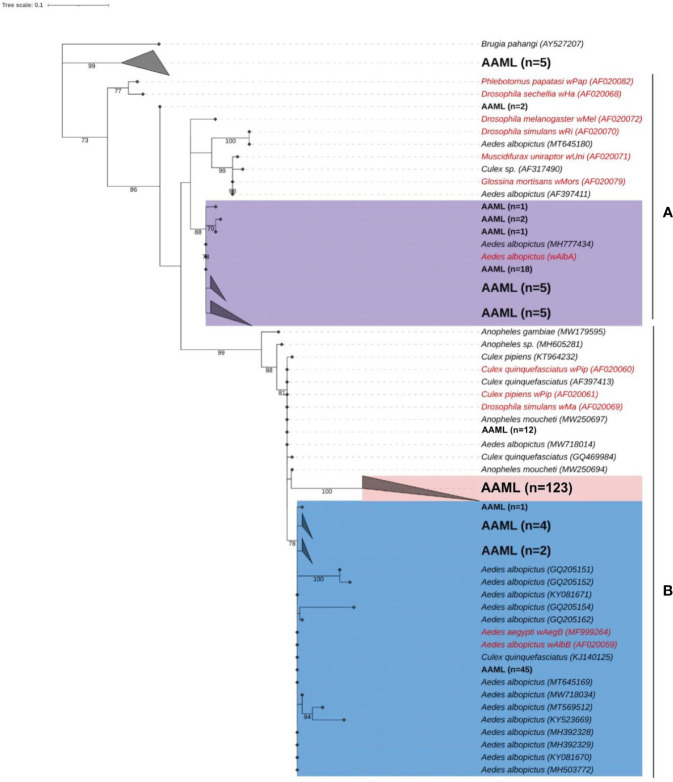
Phylogenetic analysis of *Wolbachia* according to *wsp.* Maximum-likelihood tree showing *wspAAML* sequences from *Ae. aegypti* samples collected in Metro Manila, Philippines (AAML in black bold text) and other wsp sequences obtained from other mosquito hosts (data from NCBI Blast; shown in black italicized text). Reference sequences are shown in red with an indication of the corresponding Wolbachia strains. Numbers on branches reflect the bootstrap support estimated with 1000 replications. The gray triangles are collapsed clades, each representing a node class. Ninety-eight percent of the *wspAAML* sequences clustered into either supergroup A or B. The tree shows two Wolbachia strains, namely *wAlbA* (violet) under supergroup A, *wAlbB/wAegB* (blue), and a group AAML under supergroup B (red). Scale bar indicates a phylogenetic distance of 0.1 nucleotide substitutions per site.

For phylogenetic analysis, we used 226 *wsp*AAML sequences obtained from 267 *wsp*-positive *Ae. aegypti* samples collected from Metro Manila (AAML). We excluded 41 samples due to low sequencing quality and an inability to repeat sequencing owing to an inadequate volume of DNA. According to the maximum-likelihood phylogenetic tree, 83% (187/226) of *wsp*AAML sequences in the AAML samples clustered into supergroup B, whereas only 15% (34/226) of *wsp* sequences clustered into supergroup A (**Figure 3**). The bootstrap values (≥75%; indicated on the external tree branches) supported the divergence of three clusters between supergroups A and B.

Under supergroup A, a clear cluster similar to the strain *w*AlbA was observed. Two *wsp* sequences of *Ae. aegypti* did not exhibit a clear delineation with any *Wolbachia* reference sequence. Numerous weak bootstrap support values (≤74%) were observed in deep nodes within supergroup A, indicating high genetic diversity. Under supergroup B, the *wsp*AAML sequences of *Ae. aegypti* samples were nested within *Wolbachia* cluster *w*AegB/*w*AlbB together with other *wsp* sequences derived from *Ae. albopictus* or *Culex* spp. We found 12 *wsp*AAML sequences of *Ae. aegypti* samples that fall under the *w*Pip strain. However, another cluster was solely composed of *wsp*AAML sequences found in the AAML samples (n = 123). For clarity, we hereafter refer to this AAML group (shaded in red in **Figure 3**) as the “*w*AegML” cluster. The two *Wolbachia* strains under supergroup B, i.e., *w*AegB/*w*AlbB and *w*AegML, share the same branch as *w*Pip, which was consistent with the grouping previously established by Zhou et al (Zhou et al., 1998). The 12 sequences clustering with *w*Pip share similarity with *w*AegML group and *w*AegB/*w*AlbB strain. We also noted that five *wsp* sequences from the *Ae. aegypti* samples formed a distinct cluster that did not fall under any of the supergroups considered; thus, these samples possibly belong to supergroups other than A and B.

**Figure 4:**
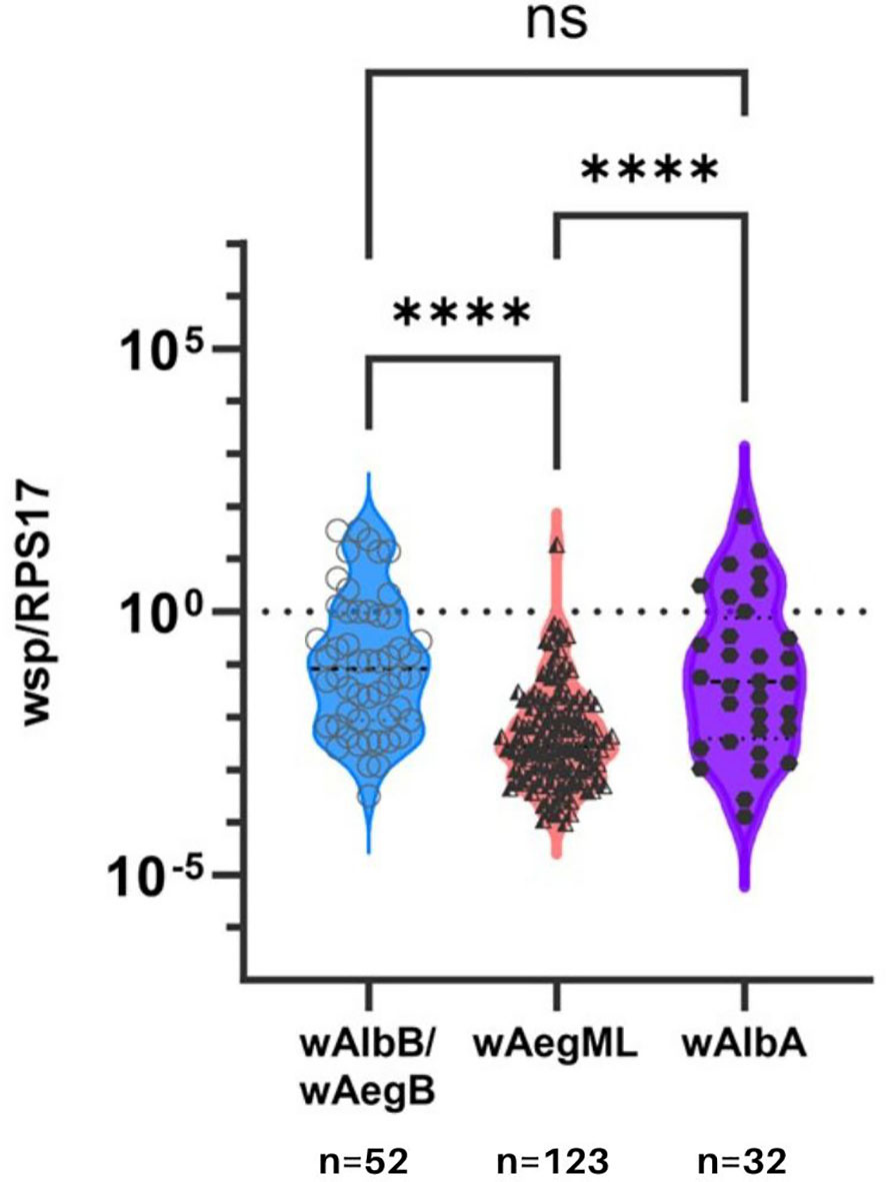
*Wolbachia* density in *Ae. aegypti* according to three identified *Wolbachia* clusters. Relative *Wolbachia* density of strains *wAlbB/wAegB*, *wAegML*, and *wAlbA* in *Ae. aegypti*. Relative density was considered the ratio of the target gene to RPS17. Values are shown on a logarithmic scale. Median *Wolbachia* relative density is indicated by a dark grey line. **** indicates p ≤0.001. ns indicates no significance between two groups.

Lastly, *Wolbachia* density of the *w*AegML group was less than either *w*AegB/*w*AlbB or *w*AlbA in *Ae. aegypti* (Dunn’s test, p <0.0001; **Figure 4**). However, *Wolbachia* density did not differ between *w*AegB/*w*AlbB and *w*AlbA strains in *Ae. aegypti* (Dunn’s test, p >0.0001).

## 4 Discussion

The presence of natural *Wolbachia* in *Ae. aegypti* has been investigated in different countries. Based on these reports, *Ae. aegypti* is either absent or when present, exists sporadically and in low prevalence (Coon et al., 2016; Teo et al., 2017; Gloria-Soria et al., 2018; Goindin et al., 2018; Thongsripong et al., 2018; Balaji et al., 2019; Bennett et al., 2019; Carvajal et al., 2019; Zhang et al., 2022; Muharromah et al., 2023). Particularly in the Philippines, *Wolbachia* has been detected in cities within Metropolitan Manila finding a relatively low prevalence for both *16S rRNA* (13.2%) and *wsp* markers (16.8%) by conventional PCR (Carvajal et al., 2019). More recently, natural *Wolbachia* detected in *Ae. aegypti* from the same mosquito population has been validated by ddRAD-sequencing. Sequence reads of 26,299 (wAlbA) and 43,778 (wAlbB) were mapped across the entire *Wolbachia* genome out of 146,239,637 filtered reads obtained (Muharromah et al., 2023). There are currently no *Wolbachia* mass release programs being implemented in the Philippines but its potential as a vector control method has been proposed (Ong et al., 2022). Hence, an economical way of conducting an initial surveillance to characterize not just the presence but also the density of natural *Wolbachia* in *Ae. aegypti* is warranted. In the present study, we used locally designed primers (*wsp*AAML) to validate the presence of natural *Wolbachia* in *Ae. aegypti* that resulted to a higher prevalence of *Wolbachia* infection in *wsp* compared to the conventional PCR method used by Carvajal et al. We also found a high detection rate of *Wolbachia*, although to a lesser extent, using an established *16S rRNA* primers. We found that relative *Wolbachia* density varied between host sex and *Wolbachia* phylogenetic groups in natural *Ae. aegypti* populations. The *Wolbachia* strains found present in *Ae. aegypti* were closely related to strains found in *Ae. albopictus*. We identified one cluster in the phylogenetic tree referred to as *w*AegML that was present in lower density but in higher prevalence in *Ae. aegypti.*

*Wolbachia* is ubiquitous in numerous host species (Noda et al., 2001; Kyei-Poku et al., 2005; Ros et al., 2009; Hughes et al., 2011) but its presence in mosquitoes vary. Presence of natural *Wolbachia* is usually high among *Culex* spp. and *Ae. albopictus* which are hosts regarded as naturally infected. On the other hand, *Anopheles gambiae* (8%-24%) and *Ae. aegypti* (4.3%-58%) exhibit variable prevalence results that differs according to geographical location (Inácio da Silva et al., 2021). In the present study, *Ae. aegypti* collected from Metro Manila, Philippines demonstrated natural *Wolbachia* prevalence of 40.0% and 62.2% when targeting *16S rRNA* and *wsp*, respectively. This finding is consistent with other studies that detected natural *Wolbachia* in *Ae. aegypti* (Coon et al., 2016; Teo et al., 2017; Thongsripong et al., 2018; Balaji et al., 2019; Bennett et al., 2019; Carvajal et al., 2019; Kulkarni et al., 2019; Muharromah et al., 2023). Given the high prevalence rate of natural *Wolbachia* detected here, we suggest that the use of location-specific primers for qPCR increased the sensitivity of our detection method.

In a previous study, our laboratory performed conventional PCR-based detection of *Wolbachia* infection in *Ae. aegypti* collected from the current study location, finding a relatively low prevalence considering both *16S rRNA* (13.2%) and *wsp* markers (16.8%). Contrastingly, a study conducted by Gloria-Soria et al. (2018) revealed the absence of *Wolbachia* in 117 *Ae. aegypti* mosquitoes collected from Cebu province, Philippines (Gloria-Soria et al., 2018). In both studies, conventional PCR was used. Although the primers used can amplify multiple *Wolbachia* strains, they were not designed using natural *Wolbachia* populations from *Ae. aegypti* mosquitoes in the local regions. Additionally, the difference in the prevalence of natural *Wolbachia* in Metropolitan Manila (Carvajal et al., 2019; Muharromah et al., 2023) and Cebu, Philippines (Gloria-Soria et al., 2018) further supports the fact that the presence of natural *Wolbachia* is variable and sporadic. The same situation occurs in China where infection prevalence between prefectures differed from 0 to 41.7% (Zhang et al., 2022).

Different *Wolbachia* primers used for PCR assays vary in terms of efficiency and coverage. The performance of 13 *Wolbachia* primer pairs was previously assessed using samples from a wide range of hosts representing supergroups A-F. The results varied, even among primers targeting the same gene, and only two primer sets yielded identical results, with other primers resulting in incorrectly sized amplifications (Simões et al., 2011). In the present study, to address the potential issue of primer incompatibility, we designed location-specific primers for *wsp* based on published sequences of *Wolbachia* populations in the same regions (Carvajal et al., 2019). In order to avoid bias, we included *16S rRNA* marker which we did not design and has already been established (Fraser et al., 2020). Using either marker demonstrated a high detection rate with *wsp*AAML being 22% higher than *16S rRNA*. This suggests that the qPCR method led to an improved natural *Wolbachia* detection rate in *Ae. aegypti* owing to primer compatibility.

Notably, the qPCR method requires the use of primers that yield short amplicons (150 bp), which may have contributed to an increase in sensitivity. Additionally, qPCR has a lower limit of detection and higher sensitivity relative to conventional PCR, which could also explain the higher detection rate in the current study (Mee et al., 2015; Xia et al., 2018). When we compared *Wolbachia* density between samples found to be *Wolbachia*-negative or positive using conventional PCR, we found a 30-fold higher median *Wolbachia* density in the *Wolbachia*-positive samples by qPCR. We also found low *Wolbachia* density. Therefore, it is likely that both the newly designed primers and qPCR helped increase the natural *Wolbachia* detection rate relative to the detection performance of conventional PCR. These results suggest that location-specific primers may help validate the presence and determine *Wolbachia* density in *Ae. aegypti* samples with transient, sporadic, and/or low density natural *Wolbachia* infection.

The use of *Ae. aegypti* mosquitoes previously subjected to *Wolbachia* detection (Carvajal et al., 2019), allowed us to define potential reasons (e.g., primer incompatibility) for the sporadic detection of natural *Wolbachia* in *Ae. aegypti*. However, the possibility of contamination of *Wolbachia* from other mosquito host species in the larval stage cannot be completely avoided and therefore should be carefully considered (Ross et al., 2020). It is recommended that a comprehensive approach including imaging-based technique (FISH, IFA), demonstration of maternal transmission, reproductive manipulation e.g., CI, and antibiotic treatment of *Wolbachia* can be used to confirm natural infection in *Ae. aegypti*. Nevertheless, an economical way to improve detection for initial surveillance is also needed considering the sporadic and low-density presence of *Wolbachia* in Philippine *Ae. aegypti*.

Meanwhile, the present study adds to the existing evidence that *Wolbachia* (*wsp*) sequences found in *Ae. aegypti* belong to either supergroup A or B. Most *wsp* sequences found in *Ae. aegypti* collected from Metro Manila, Philippines were categorized under supergroup B consistent with the previous studies that utilized known *Wolbachia* primers and ddRAD-Seq, respectively (Carvajal et al., 2019; Muharromah et al., 2023). Both supergroups are widespread among arthropods and belong to a single monophyletic lineage (Gerth et al., 2014; Zug and Hammerstein, 2015). Supergroup B is likely to be the dominant supergroup found in naturally infected *Ae. aegypti*, as reported in most previous studies (Coon et al., 2016; Balaji et al., 2019; Carvajal et al., 2019; Kulkarni et al., 2019). *Wolbachia* strains under supergroup B usually reside in their hosts at high density (Hu et al., 2020; Walker et al., 2021) and exhibit resilience to cyclical heat stress, allowing them to persist in host populations (Ross et al., 2017). In our study, *Wolbachia* density is found to be relatively low. Balaji et al. (2019) reported 1.01 and 1.76 *wsp*/*Rps17* of *Wolbachia* in male and female, respectively whereas we found an overall range of -4.02 to 1.81 regardless of gender. Natural *Wolbachia* density in *Ae. aegypti* differs throughout the developmental stage where significantly low density commonly manifests from the adult stage (Balaji et al., 2019). Our study only used random field-collected adult samples without considering age of adult mosquitoes that could account for the difference.

Interestingly, we detected three clusters representing the *Wolbachia* strains *w*AlbA, *w*AegB/*w*AlbB, and *w*AegML. Due to the inability to conduct further experiments to validate strain, we refer to another cluster as merely *w*AegML group. The bacterial density of *w*AegML was significantly lower than that of *w*AlbA, *w*AegB/*w*AlbB, and 62.8% of the *wsp* sequences in our *Ae. aegypti* samples belong to the *w*AegML. It is important to recognize that our study only used *wsp* for phylogenetic analysis which somehow limit our capacity to establish relatedness. Therefore, it is important that future studies utilize other markers and incorporate MLST data for a more accurate strain differentiation (Wang et al., 2020). Further experiments are needed to provide conclusive evidence on what impact sporadic and low-density natural *Wolbachia* in *Ae. aegypti* have.

Lastly, previous studies have revealed sex-specific *Wolbachia* density differences in natural populations of planthoppers *S. furcifera* and *L. striatellus* (Noda et al., 2001), as well as *D. citri* (Hoffmann et al., 1986)*, Drosophila* spp (Correa and Ballard, 2012), *Cx. Pipiens* (Echaubard et al., 2010), *Ae. albopictus* (Tortosa et al., 2010; Calvitti et al., 2015), and *Ae. aegypti* (Mejia et al., 2022). We found that natural *Wolbachia* density in *Ae. aegypti* males was higher than in females, which is consistent with observations in *D. citri* (Hoffmann et al., 1986) and *Ae. albopictus* (Tortosa et al., 2010). Such sex-specific variation was only previously observed in *Ae. albopictus* mosquitoes carrying the *w*AlbB strain (Tortosa et al., 2010). A recent study on *Wolbachia* density in *Ae. aegypti* also reported the consistent densities in females throughout their lifetime whereas males demonstrate relatively higher and variable *Wolbachia* densities, in testes (Mejia et al., 2022). Although the biology underlying sex-specific differences in *Wolbachia* density is unknown, biological differences between the host sexes could explain our finding. For instance, female mosquitoes tend to have an expanded microbial composition relative to that of males, resulting in more bacterial competition (Zouache et al., 2011). Female mosquitoes are hematophagous, and the composition of bacterial microbiota in mosquitoes largely depends on nutrient intake (Gaio et al., 2011; Minard et al., 2013). Thus, the digestion process of female mosquitoes may act as a barrier to the survival of some symbionts, including *Wolbachia*.

Other studies have found that females exhibit higher *Wolbachia* densities than males in *S. furcifera*, *L. striatellus* (Noda et al., 2001), *Drosophila* spp (Correa and Ballard, 2012), *Cx. pipiens* (Echaubard et al., 2010), and *Ae. albopictus* (Tortosa et al., 2010; Calvitti et al., 2015). Thus, further investigation on gender-specific effects considering other coexisting factors is warranted. Our study only explored differences of relative *Wolbachia* density between male and female adult mosquitoes. It is important to further characterize how *Wolbachia* density is affected by host sex in different stages of the mosquito life cycle. Determining an absolute *Wolbachia* density rather than a relative one will provide more accurate information. Additionally, supplementing this with microscopic evidence will demonstrate the changes in *Wolbachia* density occurring between male and female mosquitoes during the insects’ life cycle.

## 5 Conclusion

Host species in which *Wolbachia* is naturally found usually exhibit stable high prevalence, high density, and heritable infection. Nevertheless, this is not the case for *Ae. aegypti*. Our study proposes that presence of natural *Wolbachia* in *Ae. aegypti* does not mean absolute presence, rather natural *Wolbachia* in this host is sporadic, and low-density. In the present study, we focused on investigating the factors that affect such occurrence. The use of *Ae. aegypti* samples previously subjected to *Wolbachia* detection by conventional PCR and ddRAD-Seq (Carvajal et al., 2019; Muharromah et al., 2023) allowed us to explore the biological (*Wolbachia* strain and host sex) and methodological (method of detection, primer incompatibility) factors affecting *Wolbachia* prevalence rate and density. This potentially explains the varying presence of natural *Wolbachia* in this host. Our findings suggest that (i) location-specific primers can improve *Wolbachia* density detection; (ii) relative *Wolbachia* densities are influenced by host sex and bacterial strain; (iii) majority of *Wolbachia* sequences clustered into a group referred to here *w*AegML that was present at a low density in *Ae. aegypti*. Overall, designing location-specific primers is economical for initial validation considering limited resources in other countries. These factors must be accounted for when conducting initial surveillance in places where no mass release programs have been conducted (e.g., Philippines). Moving forward, a comprehensive detection of natural *Wolbachia* infection in *Ae. aegypti* using multiple methods is still warranted.

## Data availability statement

The original contributions presented in the study are included in the article/**Supplementary Material**. The generated wspAAML sequences are available in GenBank database under the Accession Numbers PP278103-PP278328. Further inquiries can be directed to the corresponding author.

## Author contributions

JR: Conceptualization, Formal Analysis, Investigation, Methodology, Writing – original draft, Writing – review & editing. TS: Data curation, Formal Analysis, Investigation, Methodology, Writing – review & editing. YS: Supervision, Writing – review & editing. KW: Conceptualization, Funding acquisition, Project administration, Resources, Supervision, Validation, Writing – review & editing.
