# Supplementary material for: Detection and quantification of natural Wolbachia in Aedes aegypti in Metropolitan Manila, Philippines using locally designed primers
Source: Front Cell Infect Microbiol. 2024 Mar 18;14:1360438. doi: 10.3389/fcimb.2024.1360438 (PMC10982481; doi:10.3389/fcimb.2024.1360438)
Supplement: Supplementary file 1 [file DataSheet_1.pdf]

Supplementary Material

1 Supplementary Data

Supplementary data shows the conventional PCR and taqman results of individual *Ae. aegypti* samples.

Sample	Place	F/M	PCR 16S	PCR WSP	Cq 16S	16S	Cq WSP	WSP	Cq RPS17
AAML1	MTLP-1	F	neg	neg	36.17	neg	37.24	neg	25.19
AAML2	PSY	F	neg	neg	32.84	pos	33.02	pos	27.39
AAML3	MTLP-1	M	neg	neg	40	neg	37.09	neg	26.95
AAML4	QZC-3	M	neg	neg	34.69	neg	34.94	neg	25.87
AAML5	QZC-3	F	neg	neg	39.63	neg	37.57	neg	27.3
AAML6	MTLP-2	F	neg	neg	34.99	neg	37.55	neg	25.06
AAML7	MTLP-1	F	neg	neg	33.02	pos	40	neg	30.9
AAML8	CAL-N	F	pos	pos	36.02	neg	40	neg	27.08
AAML9	MTLP-2	M	neg	neg	37.53	neg	34.67	neg	27.22
AAML10	CAL-N	F	neg	neg	35.56	neg	36.09	neg	25.01
AAML11	CAL-N	F	neg	neg	35.39	neg	35.75	neg	26.29
AAML12	QZC-2	M	pos	pos	27.6	pos	31.88	pos	29.01
AAML13	QZC-3	M	neg	neg	36.19	neg	35.17	neg	26.86
AAML14	QZC-1	F	neg	neg	40	neg	34.54	neg	26.29
AAML15	QZC-3	F	neg	neg	37.25	neg	35.13	neg	24.65
AAML16	LSP-1	F	neg	neg	30.96	pos	35.07	neg	26.37
AAML17	LSP-1	F	neg	neg	35.94	neg	35.54	neg	25.06
AAML18	TGG	M	neg	neg	36.86	neg	34.69	neg	28.13
AAML19	TGG	F	neg	neg	38.14	neg	34.57	neg	23.64
AAML20	TGG	M	neg	neg	36.79	neg	35.57	neg	27.88
AAML21	QZC-4	M	neg	neg	37.14	neg	35.66	neg	26.21
AAML22	CAL-N	F	neg	neg	35.67	neg	35.86	neg	24.34
AAML23	QZC-2	M	neg	neg	36.06	neg	36.03	neg	26.34
AAML24	QZC-1	M	neg	neg	32.21	pos	35.52	neg	24.76
AAML25	QZC-1	F	neg	neg	37.02	neg	36.69	neg	25.19
AAML26	LSP-1	F	neg	neg	36.29	neg	33.9	pos	23.34
AAML27	LSP-1	M	neg	neg	35.24	neg	35.36	neg	28.05
AAML28	LSP-2	F	neg	neg	40	neg	35.15	neg	24.34
AAML29	MTLP-1	F	neg	neg	36.58	neg	34.64	neg	23.79
AAML30	MTLP-2	F	neg	neg	35.67	neg	36.2	neg	25.03
AAML31	LSP-1	F	neg	neg	36.06	neg	34.95	neg	26.13
AAML32	LSP-2	M	neg	neg	32.21	pos	35.57	neg	27.81
AAML33	QZC-4	M	neg	neg	37.02	neg	35.23	neg	25.17
AAML34	QZC-4	F	neg	neg	36.29	neg	35.47	neg	23.28

AAML35	QZC-3	F	neg	neg	35.87	neg	35.38	neg	26.18
AAML36	QZC-3	M	neg	neg	33.63	pos	35.26	neg	28.72
AAML37	QZC-3	M	neg	neg	35.17	neg	34.99	neg	32.83
AAML38	MTLP-1	F	neg	neg	35.72	neg	35.27	neg	27.49
AAML39	CAL-N	M	neg	neg	34.84	neg	37.07	neg	26.36
AAML40	QZC-3	F	neg	neg	33.69	pos	33.63	pos	22.98
AAML41	QZC-1	F	neg	neg	34.35	pos	35.4	neg	24.61
AAML42	QZC-1	M	neg	neg	35.19	neg	34.76	neg	25.11
AAML43	LSP-1	F	neg	neg	35.3	neg	33.15	pos	24.9
AAML44	LSP-2	M	neg	neg	34.28	pos	34.86	neg	25.83
AAML45	LSP-2	M	neg	neg	35.44	neg	35.01	neg	28.81
AAML46	MTLP-1	F	neg	neg	35.49	neg	35.15	neg	25.38
AAML47	MTLP-2	F	neg	neg	36.99	neg	34.53	neg	25.48
AAML48	CAL-N	F	neg	neg	35.07	neg	35.29	neg	27.19
AAML49	QZC-2	F	neg	neg	35.64	neg	34.99	neg	24.03
AAML50	QZC-3	F	neg	neg	36.24	neg	36.4	neg	26.32
AAML51	QZC-1	F	neg	neg	35.19	neg	35.64	neg	25.3
AAML52	QZC-1	M	neg	neg	36.55	neg	35.99	neg	25.05
AAML53	QZC-1	M	neg	neg	35.01	neg	34.75	neg	24.79
AAML54	MTLP-2	F	neg	neg	35.14	neg	35.33	neg	26.57
AAML55	MTLP-2	F	neg	neg	35.01	neg	33.65	pos	23.24
AAML56	MTLP-2	F	neg	neg	37.18	neg	33.13	pos	25.53
AAML57	MTLP-2	F	neg	neg	34.65	neg	34.96	neg	25.41
AAML58	VAL	F	neg	neg	34.65	neg	35.04	neg	24.58
AAML59	VAL	M	neg	neg	35.37	neg	35.35	neg	23.29
AAML60	VAL	M	neg	neg	36.57	neg	35.28	neg	27.24
AAML61	VAL	F	neg	neg	34.69	neg	34.85	neg	23.19
AAML62	QZC-3	F	neg	neg	35.05	neg	36.03	neg	25.08
AAML63	PSY	F	neg	neg	36.03	neg	33.59	pos	26.77
AAML64	MTLP-1	F	neg	neg	35.7	neg	33.15	pos	25.04
AAML65	MTLP-1	F	neg	neg	35.43	neg	34.29	pos	34.16
AAML66	MTLP-1	F	neg	neg	34.71	neg	34.25	pos	25.13
AAML67	QZC-2	F	neg	neg	35.14	neg	33.96	pos	24.04
AAML68	MTLP-1	M	neg	neg	36.22	neg	35.79	neg	24.96
AAML69	QZC-3	M	neg	neg	35.42	neg	35.42	neg	28.15
AAML70	QZC-3	M	neg	neg	36.1	neg	34.95	neg	26.54
AAML71	QZC-3	M	neg	neg	36.79	neg	34.31	pos	26.65
AAML72	QZC-1	F	neg	neg	36.04	neg	34.24	pos	23.04
AAML73	QZC-1	F	neg	neg	36.88	neg	28.86	pos	24.94
AAML74	QZC-4	M	neg	neg	36.48	neg	29.14	pos	26.15
AAML75	QZC-4	F	neg	neg	35.79	neg	29.41	pos	24.76
AAML76	QZC-4	F	neg	neg	37.04	neg	30.09	pos	28.95
AAML77	QZC-4	M	neg	neg	37.02	neg	29.34	pos	28.42

AAML78	MNL-1	M	neg	neg	36.66	neg	30.57	pos	27.41
AAML79	MNL-1	F	neg	neg	35.57	neg	28.79	pos	26.61
AAML80	MKT	F	neg	neg	35.29	neg	26.53	pos	27.45
AAML81	MKT	F	neg	neg	35.81	neg	29.11	pos	27.63
AAML82	MKT	F	neg	pos	33.05	pos	28.66	pos	28.26
AAML83	QZC-4	F	neg	neg	35	neg	29.66	pos	26.84
AAML84	PSY	F	neg	neg	35.16	neg	30.33	pos	26.4
AAML85	PSG	F	neg	neg	35.82	neg	30.95	pos	26.77
AAML86	TGG	F	neg	neg	35.75	neg	31.59	pos	26.33
AAML87	MRK	M	neg	neg	35.81	neg	31.66	pos	28.57
AAML88	MNL-1	M	neg	neg	34.07	pos	32.46	pos	28.34
AAML89	MNL-1	F	neg	neg	35.12	neg	30.2	pos	27.07
AAML90	MNL-1	F	neg	neg	35.58	neg	30.52	pos	28.23
AAML91	TGG	M	neg	neg	35.08	neg	33.83	pos	25.23
AAML92	TGG	F	neg	neg	36.26	neg	31.92	pos	26.54
AAML93	TGG	F	neg	neg	35.53	neg	35.85	neg	26.04
AAML94	TGG	F	neg	neg	35.55	neg	31.3	pos	25.3
AAML95	MNL-1	M	neg	neg	31.59	pos	30.95	pos	26.61
AAML96	MNL-1	F	pos	neg	28.29	pos	31.41	pos	29.34
AAML97	MNL-1	M	neg	neg	35.15	neg	30.46	pos	27.54
AAML98	MTLP-1	M	neg	neg	36.21	neg	30.55	pos	27.62
AAML99	PSG	M	neg	neg	35.29	neg	31.76	pos	28.8
AAML100	PSG	F	neg	pos	35.44	neg	30.05	pos	30.74
AAML101	PSG	F	neg	neg	34.72	neg	31.06	pos	26.85
AAML102	PSG	F	neg	neg	37.18	neg	31.81	pos	27.02
AAML103	QZC-4	F	pos	neg	34.76	neg	36.86	neg	26.68
AAML104	QZC-4	M	neg	neg	35.52	neg	35.27	neg	26.18
AAML105	QZC-4	F	neg	neg	35.64	neg	30.59	pos	32.73
AAML106	QZC-4	M	neg	neg	36.21	neg	30.69	pos	26.12
AAML107	QZC-4	M	neg	neg	35.41	neg	31.57	pos	28.85
AAML108	MNL-1	M	neg	neg	40	neg	31.85	pos	25.38
AAML109	MNL-1	M	neg	neg	35.91	neg	31.31	pos	27.54
AAML110	MNL-1	M	neg	neg	35.32	neg	32.91	pos	31.28
AAML111	PSG	M	neg	neg	37.33	neg	35.98	neg	30.03
AAML112	MKT	F	neg	neg	35.83	neg	34.05	pos	28.98
AAML113	MNL-1	M	neg	neg	36.64	neg	31.26	pos	28.35
AAML114	QZC-5	F	neg	neg	35.23	neg	30.71	pos	26.69
AAML115	MNL-1	M	neg	neg	36.84	neg	35.56	neg	28.49
AAML116	MNL-1	M	neg	neg	35.35	neg	33.91	pos	27.21
AAML117	MNL-1	F	neg	neg	36.36	neg	33.5	pos	26.33
AAML118	QZC-4	F	neg	neg	34.57	neg	33.48	pos	26.82
AAML119	QZC-4	F	neg	neg	34.41	pos	35.53	neg	27.17
AAML120	QZC-4	F	neg	neg	34.78	neg	35.7	neg	27.56
AAML121	MNL-2	F	neg	neg	35.26	neg	30.56	pos	27.93

AAML122	MNL-2	F	neg	neg	34.63	neg	28.19	pos	26.22
AAML123	MNL-2	F	neg	neg	34.51	neg	32.04	pos	26.69
AAML124	MNL-2	M	neg	neg	34.84	neg	32.02	pos	26.47
AAML125	MNL-2	M	neg	neg	34.77	neg	33.29	pos	25.1
AAML126	MNL-2	F	neg	neg	25.91	pos	27.68	pos	31.94
AAML127	MNL-1	F	neg	neg	34.67	neg	31.77	pos	27.82
AAML128	MNL-1	F	neg	neg	34.37	pos	31.72	pos	24.47
AAML129	MNL-1	F	neg	neg	35.31	neg	30.73	pos	27.41
AAML130	MNL-1	M	neg	neg	35.19	neg	31.69	pos	25.43
AAML131	MNL-2	M	neg	neg	34.78	neg	30.5	pos	25.16
AAML132	MNL-2	M	neg	neg	34.45	neg	31.75	pos	25.47
AAML133	QZC-5	F	neg	neg	34.17	pos	32.21	pos	25.83
AAML134	QZC-5	F	neg	pos	32.71	pos	34.92	neg	25.22
AAML135	QZC-5	M	neg	neg	34.36	pos	37.24	neg	26.81
AAML136	MNL-1	F	neg	neg	33.8	pos	35.96	neg	27.38
AAML137	MNL-1	F	neg	neg	34.69	neg	30.23	pos	24.81
AAML138	MNL-1	M	neg	neg	36.69	neg	30.84	pos	25.79
AAML139	QZC-4	F	neg	neg	34.58	neg	38.31	neg	26.18
AAML140	VAL	M	neg	neg	36.05	neg	35.63	neg	28.2
AAML141	MKT	F	neg	neg	34.27	pos	29.84	pos	28.29
AAML142	MKT	F	neg	neg	33.96	pos	29.57	pos	24.14
AAML143	MKT	M	neg	neg	34.55	neg	31.9	pos	25.66
AAML144	MKT	F	neg	neg	34.52	neg	31.77	pos	24.33
AAML145	MKT	F	neg	neg	34.55	neg	30.54	pos	26.72
AAML146	MNL-2	F	neg	neg	34.66	neg	30.69	pos	24.2
AAML147	MNL-2	F	neg	neg	33.95	pos	30.59	pos	25.51
AAML148	MNL-2	F	neg	neg	35.09	neg	32	pos	26.2
AAML149	MRK	F	neg	neg	32.21	pos	33.68	pos	23.65
AAML150	CAL-S	F	neg	neg	34.99	neg	29.94	pos	24.56
AAML151	QZC-2	F	neg	neg	35.62	neg	34.16	pos	25.01
AAML152	QZC-4	M	pos	pos	29.57	pos	30.36	pos	25.16
AAML153	CAL-S	F	pos	pos	40	neg	27.15	pos	25.32
AAML154	QZC-5	M	pos	pos	25.52	pos	26.51	pos	26.52
AAML155	QZC-5	F	pos	pos	23.04	pos	25.76	pos	27.2
AAML156	CAL-N	F	pos	pos	28.47	pos	31.67	pos	24.19
AAML157	CAL-N	F	neg	neg	31.52	pos	32.17	pos	22.6
AAML158	CAL-N	M	neg	neg	32.58	pos	34.12	pos	24.23
AAML159	QZC-3	F	neg	neg	32.93	pos	35.3	neg	24.62
AAML160	QZC-3	M	neg	neg	31	pos	31.35	pos	27.02
AAML161	QZC-3	F	pos	pos	23.41	pos	24.46	pos	26.89
AAML162	QZC-3	M	neg	neg	33.05	pos	34.25	pos	25.52
AAML163	QZC-2	M	neg	neg	35.16	neg	34.17	pos	26.32
AAML164	QZC-3	M	pos	pos	29.82	pos	30.72	pos	25.12

AAML165	CAL-S	M	pos	pos	28.16	pos	28.55	pos	25.62
AAML166	CAL-S	F	pos	pos	28.17	pos	28.3	pos	24.61
AAML167	CAL-N	F	neg	neg	30.3	pos	30.05	pos	25.91
AAML168	CAL-S	F	neg	neg	33.03	pos	33.39	pos	25.2
AAML169	QZC-3	F	neg	neg	33.3	pos	35.6	neg	24.92
AAML170	QZC-1	M	neg	neg	32.23	pos	32.37	pos	25.14
AAML171	CAL-N	F	neg	neg	30.94	pos	31.84	pos	25.52
AAML172	CAL-S	F	neg	neg	29.06	pos	31.19	pos	23.25
AAML173	QZC-2	M	neg	neg	30.78	pos	31.59	pos	26.24
AAML174	QZC-3	F	neg	neg	32.58	pos	33.1	pos	24.36
AAML175	QZC-2	M	pos	pos	32.19	pos	33.56	pos	25.67
AAML176	QZC-2	F	pos	pos	29.55	pos	30.87	pos	24.52
AAML177	QZC-2	M	pos	pos	30.24	pos	31.03	pos	25.96
AAML178	QZC-4	M	pos	pos	26.9	pos	27.11	pos	25.36
AAML179	QZC-2	F	pos	pos	28.35	pos	29.34	pos	25.24
AAML180	QZC-3	F	neg	neg	35.54	neg	36.14	neg	25.33
AAML181	CAL-N	M	neg	neg	32.44	pos	28.2	pos	24.54
AAML182	CAL-N	M	neg	neg	31.47	pos	32.39	pos	23.45
AAML183	CAL-N	M	neg	neg	29.33	pos	31.3	pos	25.67
AAML184	QZC-4	F	pos	pos	25.57	pos	26.7	pos	25.17
AAML185	QZC-2	F	neg	neg	32.56	pos	30.58	pos	23.23
AAML186	QZC-2	F	neg	neg	36.46	neg	35.03	neg	24.8
AAML187	QZC-2	F	pos	pos	33.64	pos	33.99	pos	25.57
AAML188	QZC-3	F	pos	pos	30.95	pos	31.8	pos	25.2
AAML189	QZC-4	F	neg	neg	31.72	pos	31.91	pos	26.02
AAML190	QZC-4	F	neg	neg	30.02	pos	30.6	pos	23.07
AAML191	CAL-S	F	neg	neg	28.4	pos	34.98	neg	23.06
AAML192	CAL-S	F	neg	neg	29.62	pos	34.68	neg	23.06
AAML193	QZC-3	F	neg	neg	31.92	pos	34.17	pos	24.23
AAML194	QZC-3	F	neg	neg	32.32	pos	34.41	pos	23.4
AAML195	QZC-4	M	neg	neg	30.09	pos	31.41	pos	24.83
AAML196	CAL-N	F	neg	neg	35.65	neg	28.73	pos	26.89
AAML197	QZC-3	F	neg	neg	34.16	pos	31.07	pos	24.02
AAML198	QZC-2	M	neg	neg	33.26	pos	33.91	pos	25.21
AAML199	CAL-N	F	neg	neg	33.98	pos	33.91	pos	24.07
AAML200	QZC-2	F	neg	neg	33.46	pos	32.4	pos	25.89
AAML201	CAL-N	F	neg	neg	34.03	pos	33.37	pos	26.52
AAML202	CAL-N	F	neg	neg	35.86	neg	35.55	neg	25.35
AAML203	CAL-N	F	neg	neg	34.91	neg	35.25	neg	24.91
AAML204	CAL-N	F	neg	neg	31.38	pos	34.27	pos	23.48
AAML205	CAL-N	M	neg	neg	40	neg	40	neg	24.29
AAML206	QZC-3	F	neg	neg	32.26	pos	33.25	pos	22.11
AAML207	CAL-N	M	neg	neg	29.67	pos	30.41	pos	23.98
AAML208	CAL-S	M	neg	neg	33.67	pos	30.29	pos	22.37

AAML209	QZC-2	F	neg	neg	35.14	neg	33.27	pos	23.2
AAML210	QZC-3	M	neg	neg	35.06	neg	38.69	neg	25.27
AAML211	QZC-1	F	neg	neg	34.5	neg	35.73	neg	24.28
AAML212	CAL-S	F	neg	neg	35.48	neg	34.06	pos	24.39
AAML213	CAL-S	F	neg	neg	38.08	neg	36	neg	23.68
AAML214	CAL-N	F	neg	neg	35.36	neg	33.09	pos	23.06
AAML215	QZC-2	F	neg	neg	32.55	pos	32.65	pos	24.79
AAML216	QZC-3	F	neg	neg	31.52	pos	32.27	pos	22.55
AAML217	QZC-3	F	neg	neg	35.9	neg	34.59	neg	22.33
AAML218	VAL	F	neg	neg	40	neg	34.22	pos	22.72
AAML219	QZC-2	M	neg	neg	34.9	neg	34.67	neg	22.63
AAML220	QZC-1	F	neg	neg	40	neg	35.16	neg	24.11
AAML221	QZC-1	F	neg	neg	40	neg	34.56	neg	21.85
AAML222	QZC-3	F	neg	neg	35.95	neg	32.32	pos	22.79
AAML223	CAL-N	F	neg	neg	37.14	neg	35.12	neg	21.79
AAML224	QZC-2	F	neg	neg	40	neg	35.38	neg	23.73
AAML225	PRNQ	F	neg	neg	32.55	pos	30.99	pos	23.51
AAML226	PRNQ	F	neg	neg	35.03	neg	33.81	pos	25.29
AAML227	PRNQ	M	neg	neg	35.51	neg	34.37	pos	25.8
AAML228	MTLP-2	F	pos	pos	31.58	pos	32.19	pos	23.09
AAML229	MTLP-1	F	neg	neg	29.75	pos	30.16	pos	23.22
AAML230	QZC-2	F	neg	neg	40	neg	28.98	pos	26.56
AAML231	LSP-1	F	neg	neg	32.32	pos	32.06	pos	23.25
AAML232	CAL-N	M	neg	pos	24.04	pos	25.23	pos	31.24
AAML233	PSY	F	neg	neg	34.31	pos	31.71	pos	23.21
AAML234	MTLP-2	M	neg	pos	29.85	pos	31.76	pos	27.12
AAML235	PSY	F	pos	pos	29.78	pos	32.52	pos	23.29
AAML236	MTLP-2	F	pos	pos	22.22	pos	29.41	pos	27.04
AAML237	MTLP-2	F	pos	pos	28.11	pos	31.16	pos	24.91
AAML238	MTLP-2	M	neg	neg	29.54	pos	29.26	pos	25.38
AAML239	LSP-1	M	neg	neg	31.79	pos	25.42	pos	24.45
AAML240	LSP-1	M	pos	pos	31.22	pos	28.61	pos	25.68
AAML241	LSP-1	M	neg	pos	32.85	pos	33.13	pos	26.28
AAML242	MTLP-1	M	neg	neg	35.59	neg	33.31	pos	25.93
AAML243	PRNQ	M	neg	neg	31.1	pos	33.14	pos	24.13
AAML244	MTLP-2	F	pos	pos	32.23	pos	36.11	neg	24.27
AAML245	PRNQ	M	pos	pos	30.26	pos	33.51	pos	23.8
AAML246	MTLP-2	F	neg	neg	32.35	pos	33.56	pos	24.83
AAML247	TGG	M	pos	pos	30.36	pos	31.89	pos	32.52
AAML248	PSY	M	neg	neg	35.51	neg	31.36	pos	25.55
AAML249	PSY	F	pos	pos	23.83	pos	25.22	pos	26.86
AAML250	MTLP-2	F	neg	neg	29.96	pos	32.86	pos	23.66
AAML251	PRNQ	M	neg	neg	36.23	neg	35.22	neg	26.09

AAML252	PRNQ	M	neg	pos	32.71	pos	32.84	pos	23.91
AAML253	MTLP-2	F	pos	neg	28.39	pos	29.43	pos	27.75
AAML254	MTLP-2	F	neg	pos	33.48	pos	33.66	pos	24.19
AAML255	MTLP-2	F	neg	neg	33.29	pos	33.02	pos	24.01
AAML256	PSY	F	neg	neg	27.65	pos	25.03	pos	23.52
AAML257	MTLP-1	M	neg	neg	29.13	pos	29.4	pos	23.65
AAML258	TGG	F	neg	neg	36.22	neg	36.24	neg	23.22
AAML259	MTLP-2	F	neg	neg	33.04	pos	33.8	pos	22.85
AAML260	MTLP-2	F	neg	neg	32.79	pos	31.97	pos	23.5
AAML261	MTLP-1	M	neg	pos	25.44	pos	26.12	pos	27.21
AAML262	PRNQ	M	neg	pos	24.7	pos	25.4	pos	29.27
AAML263	MTLP-2	M	pos	pos	28.22	pos	28.09	pos	25.27
AAML264	VAL	F	neg	neg	26.83	pos	26.52	pos	25.18
AAML265	PRNQ	F	neg	neg	34.82	neg	34.77	neg	23.65
AAML266	MTLP-2	F	neg	neg	30.23	pos	29.14	pos	23.6
AAML267	PSY	M	neg	neg	31.45	pos	30.81	pos	25.41
AAML268	PSG	M	neg	neg	36.63	neg	30.72	pos	22.77
AAML269	MNL-2	M	neg	neg	32.97	pos	34.78	neg	23.43
AAML270	PSG	M	neg	neg	32.67	pos	28.7	pos	22.64
AAML271	MKT	M	neg	neg	33.8	pos	31.16	pos	22.51
AAML272	MNL-1	F	neg	neg	36.15	neg	31.93	pos	24.67
AAML273	SJ-MND	M	neg	neg	34.88	neg	30	pos	26.58
AAML274	MNL-1	F	neg	neg	32.69	pos	31.72	pos	21.02
AAML275	PSY	F	neg	neg	31.74	pos	30.81	pos	23.05
AAML276	MTLP-1	F	neg	neg	39.89	neg	30.44	pos	21.75
AAML277	PSY	M	neg	neg	38.14	neg	38.18	neg	24.36
AAML278	MNL-1	M	neg	neg	36.2	neg	36.92	neg	24.69
AAML279	PSY	F	neg	neg	35.33	neg	30.97	pos	22.03
AAML280	PSG	M	neg	neg	40	neg	40	neg	24.45
AAML281	MNL-1	M	neg	neg	34.51	neg	30.79	pos	23.84
AAML282	PSY	F	neg	neg	34.13	pos	32.5	pos	23.82
AAML283	SJ-MND	M	neg	neg	35.27	neg	32.51	pos	25.58
AAML284	PSY	M	neg	neg	35.54	neg	32.1	pos	24.16
AAML285	PSY	F	neg	neg	34.95	neg	36.69	neg	23.06
AAML286	MKT	F	neg	neg	34.34	pos	34.22	pos	23.43
AAML287	MKT	M	neg	neg	36.52	neg	33.47	pos	24.65
AAML288	MKT	M	neg	neg	35.44	neg	31.41	pos	24.23
AAML289	MNL-1	F	neg	neg	37.61	neg	33.28	pos	22.59
AAML290	QZC-5	M	neg	neg	40	neg	33.41	pos	24.82
AAML291	MKT	F	neg	neg	35.81	neg	34.87	neg	21.19
AAML292	QZC-4	F	neg	neg	36.1	neg	34.15	pos	22.57
AAML293	MKT	M	neg	neg	40	neg	33.56	pos	24.49
AAML294	MNL-2	F	neg	neg	37.59	neg	34.88	neg	20.99
AAML295	SJ-MND	M	neg	neg	37.21	neg	34.74	neg	23

AAML296	MNL-2	F	neg	neg	40	neg	33.55	pos	22.23
AAML297	MRK	F	neg	neg	40	neg	35.29	neg	21.32
AAML298	MRK	F	neg	neg	40	neg	33.81	pos	21.57
AAML299	SJ-MND	F	neg	neg	40	neg	32.6	pos	21.98
AAML300	PSY	F	neg	neg	40	neg	33.13	pos	22.64
AAML301	QZC-5	F	neg	neg	40	neg	33.89	pos	20.53
AAML302	MRK	M	neg	neg	34.06	pos	33.03	pos	22.2
AAML303	MRK	F	neg	neg	35.42	neg	35.24	neg	20.75
AAML304	MNL-2	F	neg	neg	34.22	pos	37.83	neg	22.1
AAML305	QZC-4	F	neg	neg	40	neg	36.05	neg	21.53
AAML306	QZC-4	F	neg	neg	33.89	pos	34.81	neg	21.2
AAML307	QZC-4	M	neg	pos	23.79	pos	25.6	pos	30.74
AAML308	MNL-2	M	neg	neg	36.81	neg	35.42	neg	23.76
AAML309	MNL-2	F	neg	neg	35.85	neg	33.88	pos	21.1
AAML310	QZC-4	M	neg	neg	35.52	neg	35.01	neg	24.73
AAML311	QZC-4	F	neg	neg	29.91	pos	32.66	pos	20.72
AAML312	MNL-2	M	neg	neg	34.8	neg	33.9	pos	25.16
AAML313	MNL-2	M	neg	neg	38.89	neg	34.93	neg	24.47
AAML314	MNL-1	M	pos	neg	32.72	pos	32.2	pos	22.61
AAML315	MKT	M	neg	neg	40	neg	33.89	pos	23.69
AAML316	QZC-4	M	neg	neg	40	neg	35.49	neg	21.17
AAML317	MNL-2	F	neg	neg	40	neg	32.63	pos	20.42
AAML318	MNL-2	F	neg	pos	24.35	pos	25.43	pos	28.42
AAML319	QZC-5	M	neg	neg	33.93	pos	33.16	pos	22.27
AAML320	MNL-1	M	neg	neg	40	neg	34.34	pos	23.88
AAML321	MNL-1	M	neg	neg	37.93	neg	35.72	neg	22.87
AAML322	MKT	F	neg	neg	37.73	neg	34.01	pos	21.2
AAML323	QZC-5	F	neg	neg	34.44	pos	30.19	pos	21.67
AAML324	MNL-2	M	neg	neg	30.33	pos	31.77	pos	24.59
AAML325	MNL-2	F	neg	neg	28.69	pos	31.13	pos	22.5
AAML326	MKT	M	neg	neg	35.04	neg	32.52	pos	23.74
AAML327	SJ-MND	M	neg	neg	40	neg	28.86	pos	24.71
AAML328	SJ-MND	F	neg	neg	40	neg	33.19	pos	20.98
AAML329	MNL-2	F	neg	neg	40	neg	37.24	neg	22.11
AAML330	PSY	M	neg	neg	38.6	neg	34.33	pos	22.61
AAML331	PSY	F	neg	neg	40	neg	33.62	pos	21
AAML332	PSY	M	neg	pos	26.52	pos	25.88	pos	27
AAML333	MNL-2	F	neg	neg	35.54	neg	33.54	pos	23.75
AAML334	MNL-2	F	neg	neg	38.86	neg	33.78	pos	29.23
AAML335	SJ-MND	F	neg	neg	29.85	pos	32.05	pos	22.65
AAML336	MNL-2	M	pos	pos	19.16	pos	26.64	pos	23.63
AAML337	MRK	F	neg	neg	40	neg	33.33	neg	21.62
AAML338	MNL-2	M	neg	neg	36.63	neg	31.39	pos	24.12

AAML339	QZC-5	F	neg	neg	37.51	neg	33.86	pos	21.91
AAML340	QZC-4	M	neg	neg	40	neg	33.12	pos	23.17
AAML341	MNL-2	F	neg	neg	34.21	pos	35.42	neg	21.9
AAML342	MKT	M	pos	pos	34.69	neg	33.14	pos	24.49
AAML343	MNL-2	M	neg	neg	36.66	neg	36.88	neg	23.89
AAML344	MKT	M	neg	neg	37.15	neg	34.2	pos	22.76
AAML345	QZC-5	F	neg	neg	36.77	neg	40	neg	21.45
AAML346	MNL-1	M	pos	pos	24.58	pos	24.4	pos	23.63
AAML347	QZC-4	F	neg	neg	35.35	neg	38.82	neg	23.47
AAML348	QZC-5	F	neg	neg	37.39	neg	38.77	neg	22.4
AAML349	QZC-4	M	neg	neg	37.1	neg	31	pos	23.57
AAML350	QZC-4	F	neg	neg	35.92	neg	37.32	neg	22.33
AAML351	MNL-2	F	neg	neg	37.91	neg	34	pos	20.85
AAML352	MNL-2	F	neg	neg	40	neg	28.19	pos	23
AAML353	MNL-2	M	neg	neg	36	neg	40	neg	24.91
AAML354	MNL-2	F	neg	neg	35.1	neg	35.44	neg	22.34
AAML355	QZC-5	F	neg	neg	35.48	neg	36.32	neg	22.06
AAML356	PRNQ	M	neg	neg	37.56	neg	35.85	neg	23.8
AAML357	PRNQ	M	neg	neg	40	neg	36.14	neg	23.55
AAML358	PRNQ	F	neg	neg	31.39	pos	32.75	pos	21.75
AAML359	PRNQ	F	neg	neg	32.45	pos	40	neg	23.82
AAML360	PRNQ	F	neg	neg	38.14	neg	32.83	pos	21.41
AAML361	QZC-2	F	neg	neg	39.86	neg	36.94	neg	24.3
AAML362	PSY	F	neg	neg	29.58	pos	30.33	pos	24.48
AAML363	PSY	F	neg	neg	40	neg	36.46	neg	25.16
AAML364	MRK	M	neg	neg	37.02	neg	36.79	neg	25.02
AAML365	QZC-5	F	neg	neg	35.97	neg	35.51	neg	23.13
AAML366	MTLP-2	M	neg	neg	37.41	neg	35.02	neg	22.32
AAML367	PRNQ	F	neg	neg	36.8	neg	36.41	neg	22.32
AAML368	CAL-N	F	neg	neg	40	neg	35.2	neg	22.99
AAML369	CAL-N	M	neg	neg	27.72	pos	29.22	pos	25.29
AAML370	CAL-N	F	neg	neg	29.17	pos	32.87	pos	23.08
AAML371	MTLP-2	M	neg	neg	33.24	pos	34.79	neg	25.41
AAML372	MRK	M	neg	neg	34.57	neg	34.81	neg	23.92
AAML373	PRNQ	F	neg	neg	34.86	neg	32.66	pos	21.96
AAML374	MTLP-1	M	neg	pos	23.07	pos	24.62	pos	28.45
AAML375	MTLP-1	M	pos	pos	24.58	pos	24.83	pos	28.64
AAML376	MRK	F	neg	neg	35.71	neg	36.33	neg	22.56
AAML377	PRNQ	M	neg	neg	40	neg	36.41	neg	24.13
AAML378	MTLP-1	F	neg	neg	37.09	neg	35.61	neg	23.05
AAML379	LSP-2	M	neg	neg	40	neg	32.34	pos	23.54
AAML380	CAL-N	F	pos	pos	31.45	pos	33.12	pos	24.94
AAML381	CAL-S	M	neg	neg	40	neg	33.83	pos	22.99
AAML382	LSP-2	M	neg	neg	35.54	neg	35.71	neg	25.55

AAML383	LSP-2	M	neg	neg	33.92	pos	34.83	neg	22.88
AAML384	QZC-1	F	neg	neg	29.86	pos	34.81	neg	24
AAML385	MRK	F	neg	neg	35.04	neg	37.54	neg	23.13
AAML386	MRK	M	neg	neg	34.13	pos	33.72	pos	23.22
AAML387	PSG	M	neg	neg	34.04	pos	34.37	pos	23.08
AAML388	CAL-N	F	neg	neg	40	neg	38.29	neg	23.7
AAML389	QZC-1	M	neg	neg	40	neg	36.1	neg	24.04
AAML390	CAL-N	F	neg	neg	37.45	neg	35.95	neg	23.72
AAML391	CAL-N	M	pos	pos	27.8	pos	27.33	pos	26.58
AAML392	CAL-S	F	neg	neg	32.84	pos	36.55	neg	23.19
AAML393	CAL-S	M	pos	pos	23.88	pos	25.07	pos	29.62
AAML394	PRNQ	F	neg	pos	31.05	pos	32.71	pos	24.79
AAML395	QZC-5	F	neg	neg	30.57	pos	33.36	pos	22.72
AAML396	QZC-4	F	neg	neg	33.62	pos	33.7	pos	23.66
AAML397	MRK	F	neg	neg	40	neg	34.94	neg	25.31
AAML398	QZC-4	M	neg	neg	35.69	neg	35.41	neg	22.58
AAML399	MTLP-1	F	neg	neg	36.54	neg	35.67	neg	21.7
AAML400	QZC-5	F	neg	neg	33.86	pos	37.9	neg	21.75
AAML401	QZC-5	F	neg	neg	34.04	pos	35.84	neg	22.54
AAML402	QZC-2	F	neg	neg	32.74	pos	33.28	pos	22.61
AAML403	QZC-2	F	neg	pos	25.47	pos	26.61	pos	26.28
AAML404	LSP-1	F	neg	neg	33.06	pos	34.79	neg	22.69
AAML405	LSP-1	F	pos	pos	25.29	pos	27.44	pos	23.32
AAML406	LSP-1	F	pos	pos	31.15	pos	28.5	pos	24.03
AAML407	CAL-N	M	neg	neg	29.92	pos	34.67	neg	24.99
AAML408	CAL-N	M	neg	neg	40	neg	32.7	pos	23.8
AAML409	CAL-N	F	neg	neg	33.16	pos	33.72	pos	22.62
AAML410	CAL-N	M	neg	neg	35.17	neg	36.5	neg	23.85
AAML411	CAL-N	F	neg	neg	34.8	neg	35.43	neg	21.18
AAML412	CAL-N	F	neg	neg	35.14	neg	37.26	neg	27.05
AAML413	CAL-N	F	neg	neg	35.27	neg	36.25	neg	24.09
AAML414	CAL-N	M	neg	neg	31.5	pos	33.46	pos	25.35
AAML415	CAL-N	F	neg	neg	28.51	pos	29.58	pos	22.18
AAML416	CAL-N	F	neg	pos	25.81	pos	28.52	pos	28.33
AAML417	CAL-N	M	neg	neg	29.29	pos	32.47	pos	23.2
AAML418	CAL-N	M	pos	pos	24.2	pos	25.45	pos	28.02
AAML419	CAL-N	M	pos	pos	25.22	pos	25.78	pos	26.15
AAML420	QZC-4	F	neg	neg	31.52	pos	32.37	pos	21.4
AAML421	MTLP-1	F	neg	neg	34.55	neg	36.22	neg	22.51
AAML422	QZC-1	M	neg	neg	35.05	neg	37.71	neg	24.44
AAML423	QZC-1	M	neg	neg	40	neg	36.29	neg	26.4
AAML424	QZC-1	M	neg	neg	40	neg	37.59	neg	28.14
AAML425	LSP-1	M	neg	neg	34.07	pos	35.84	neg	23.15

AAML426	LSP-1	F	neg	neg	31.62	pos	34.13	pos	21.43
AAML427	QZC-5	M	neg	neg	31.29	pos	33.15	pos	21.58
AAML428	VAL	F	neg	neg	34.62	neg	40	neg	24.8
AAML429	MNL-2	F	neg	neg	37.62	neg	35.51	neg	22.11

2 Supplementary Figures/Tables

2.1 Supplementary Table 1

Name	Forward	Reverse	Probe	Amplicon size
wsp AAML01	GGAGCTCGTTATTTCGGTTCT	TGCACCAACAGTGCTGTAA	ACTGCTGCAGCAAGCAAAGACAAG	105 bp
wsp AAML02	GGTTGATGTTGAAGGGCTTTATTC	ACTAGCCCTGAAATTGCTGTTA	AGGTGCAGCATTTAACCCAGATACTGT	108 bp
wsp AAML03	AGGCCACAGACATTCATAATCC	GCTGTGAATAAAGCCCTTCAAC	TATAGCTGGTGGTGGTGCATTTGGT	111 bp
wsp AAML04	GTGGTGGTGCATTTGGTTAC	CTGCAACAGTATCTGGGTTAAATG	ACATCAGGGTTGATGTTGAAGGGCT	117 bp
wsp AAML05	AGCATCTTTTATGGCTGGTGG	AATGCTGCCACACTGTTTGC	ACGACGTTGGTGGTGCAACATTTGC	150 bp

Supplementary Table 1. The table shows the initial list of *wsp* primers and probes generated through PrimerBLAST.

2.2 Supplementary Figure 1

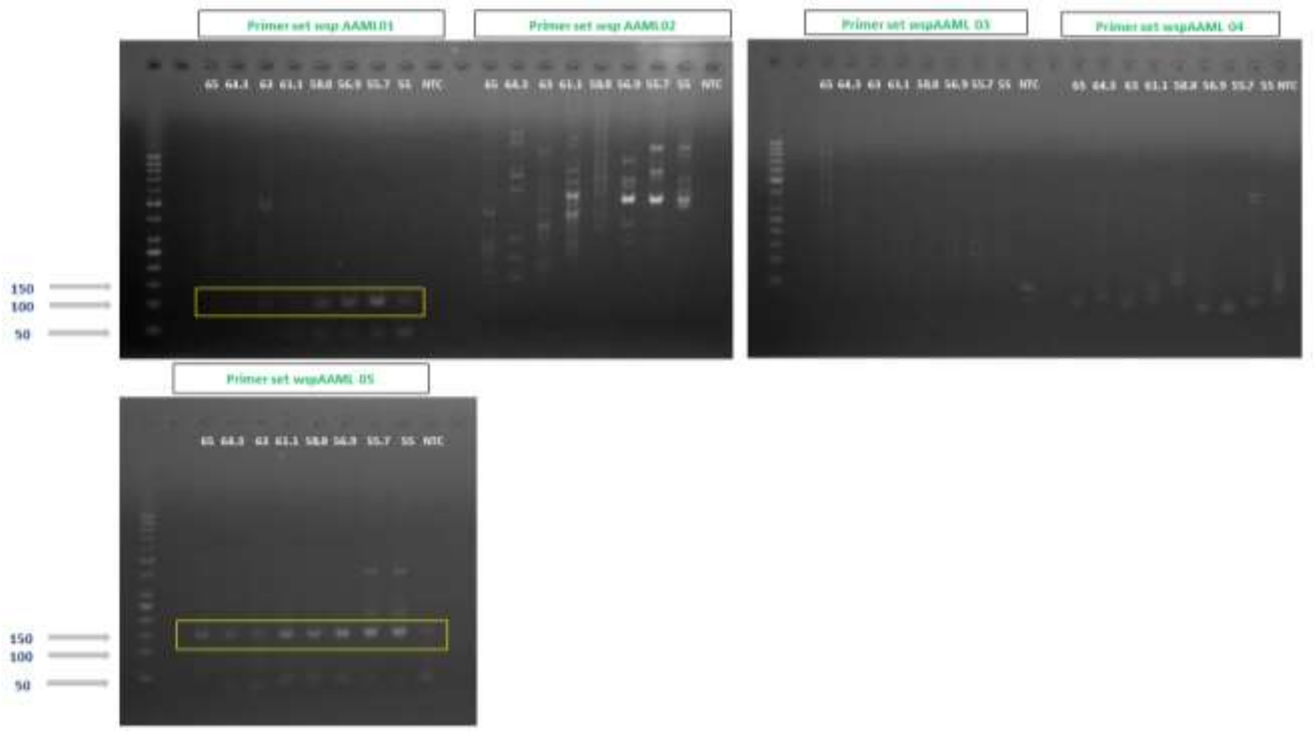

Supplementary Figure 2. The figure shows the gel electrophoresis results of the gradient PCR done for initial screening of 5 wsp primer pairs. Primer sets *wsp* AAML 01 and 05 showed correct

amplicon product size. For the NTC, only one was included in the PCR run with annealing temperature of 65°C.

2.3 Supplementary Figure 2

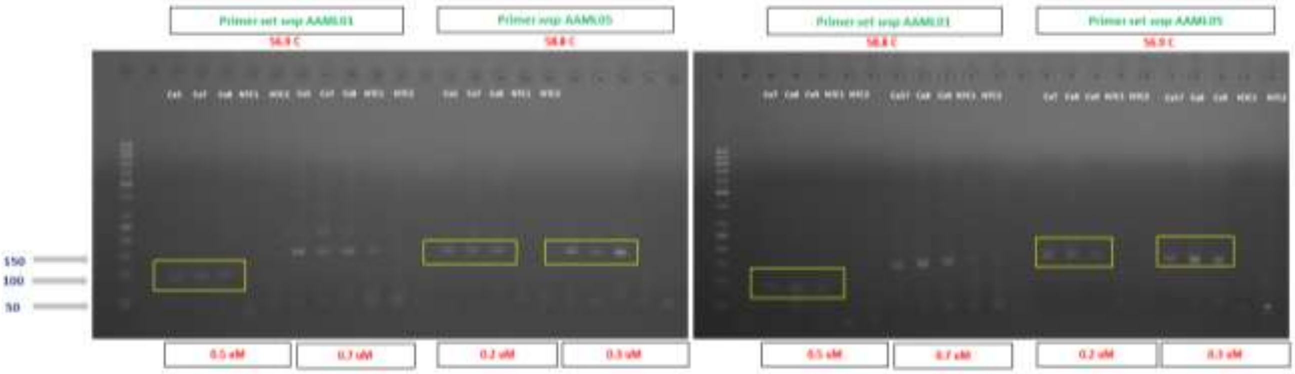

Supplementary Figure 2. The figure shows the gel electrophoresis results of primer sets *wsp* AAML 01 and 05 that previously showed the correct amplicon size in gradient PCR screening. In this experiment, different primer concentrations and two annealing temperatures were tested. Primer set *wsp* AAML 05 consistently showed correct amplicon size and did not show any amplification in NTCs.

2.4 Supplementary Figure 3

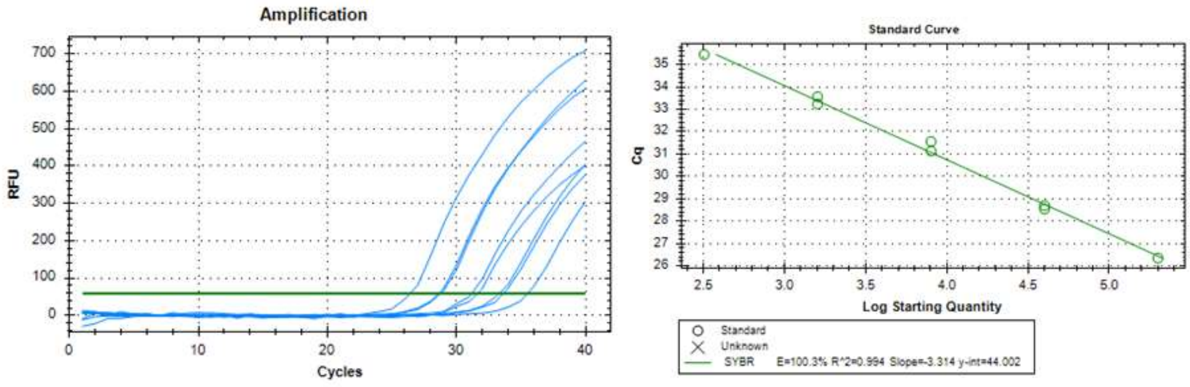

Supplementary Figure 3. The figure shows the PCR efficiency of primer set *wsp* AAML 05 of 100.3% and R^2 value equal to 0.994 using a positive control serially diluted in ten-fold.

2.5 Supplementary Figure 4

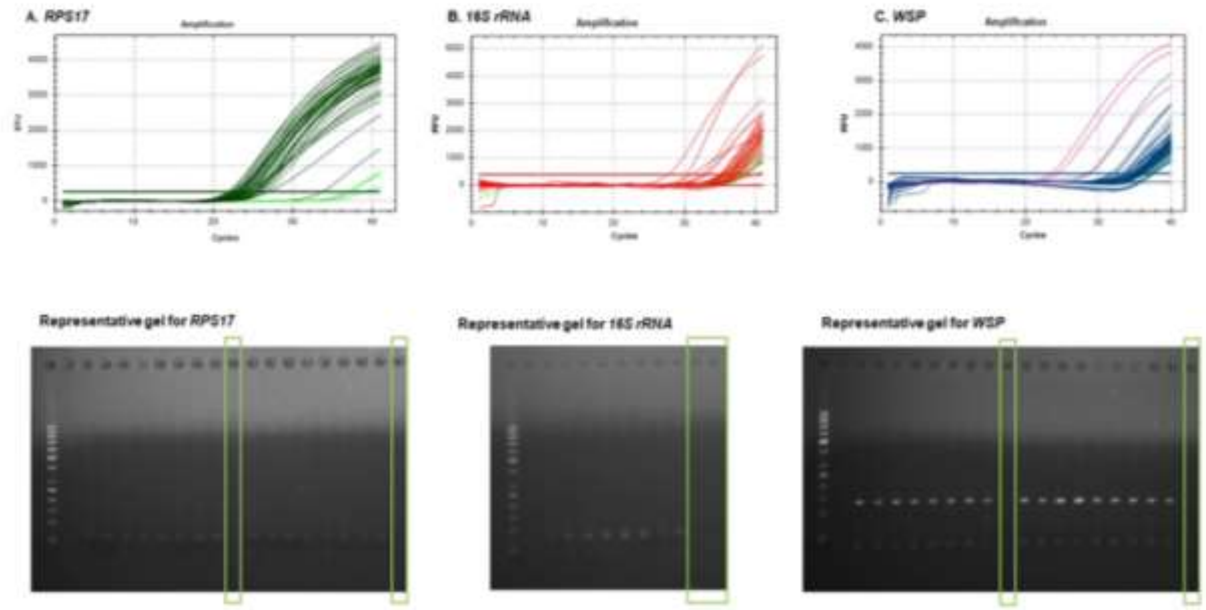

Supplementary Figure 4. The figure shows representative taqman qPCR amplification plots of each target gene and gel electrophoresis results showing no NTC amplification bands using actual *Ae. aegytpi* samples.
